# Directed Oxidative Folding of Disulfide-Rich Peptides (DRPs) Using a Pseudoproline Unit: Ion Mobility-Mass Spectrometry (IM-MS) Reveals the Conformational Landscape of the DRP

**DOI:** 10.1021/acs.analchem.6c02816

**Published:** 2026-09-17

**Authors:** Declan T. Cook, Kish R. Adoni, Konstantinos Thalassinos, Alethea B. Tabor

**Affiliations:** † Department of Chemistry, University College London, 20 Gordon Street, London WC1H 0AJ, U.K.; ‡ Department of Structural and Molecular Biology, University College London, London WC1E 6BT, U.K.; § Division of Biosciences, University College London, London WC1E 6BT, U.K.; ∥ Institute of Structural and Molecular Biology, Birkbeck College, School of Natural Sciences, University of London, London WC1E 7HX, U.K.

## Abstract

Disulfide-rich peptides (DRPs) are attractive drug development scaffolds due to their exceptional stability and specificity. Regioselective disulfide bond formation is often achieved *via* orthogonal Cys protecting groups or strategic Pro placement. However, Cys protection strategies are labor-intensive and low-yielding, while strategic Pro placement imposes sequence constraints. To broaden the chemical diversity of DRPs, we developed an alternative approach using an acid-stable oxazolidine pseudoproline to guide disulfide formation in a multicyclic model peptide. This pseudoproline was incorporated as a monomer during automated Fmoc solid-phase peptide synthesis (SPPS), without pseudoproline dipeptide synthesis. The pseudoproline remained intact under standard peptide synthesis cleavage conditions (95% TFA), allowing it to direct peptide folding. Oxidation of the cleaved peptide strongly favored one regioisomer, Cys­(I-IV)/Cys­(II-III), with 86% selectivity. Ion mobility-mass spectrometry (IM-MS) revealed that this regioisomer exhibited two dominant conformational families. Experiments involving multiple rounds of IM separation and collision activation (CA) (IM-CA-IM) showed that these families interconvert in the gas phase, likely *via cis*/*trans* isomerization at the pseudoproline amide bond. This study provides an alternative to Pro for directing disulfide bond formation, relevant to medicinal chemistry projects. It also demonstrates the power of IM-MS to characterize DRP conformational landscapes and dynamics.

## Graphical abstract

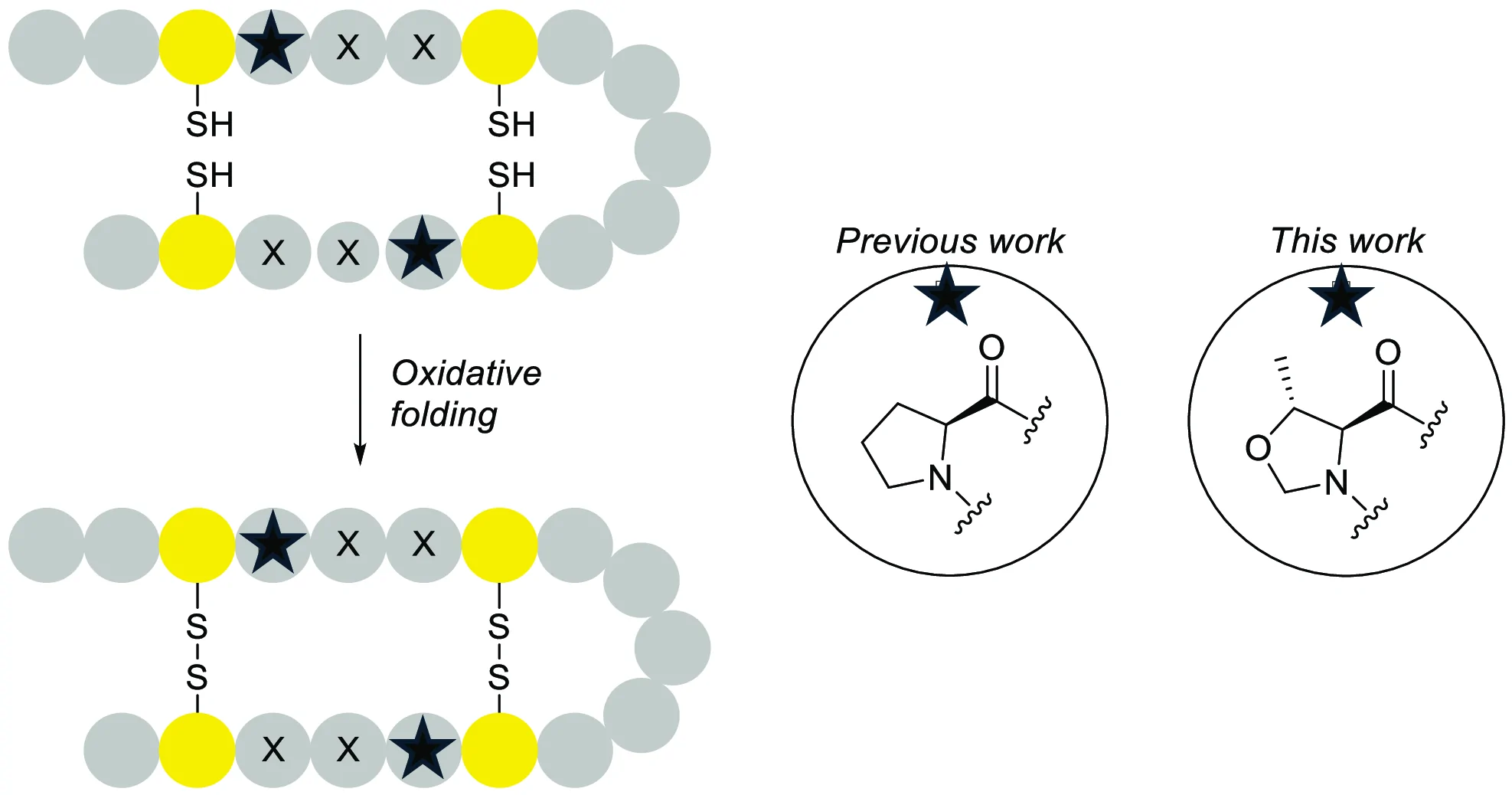

## Introduction

Disulfide-rich peptides (DRPs) are compact, cystine-stabilized molecules that display exceptional stability to thermal and enzymatic degradation.^1–3^ DRPs often exhibit high binding specificity and potency toward a broad range of targets, including proteases, ion channels, and GPCRs. Bioactive DRP scaffolds found in nature, such as the inhibitory cystine knot (ICK) peptides and cyclotides, have been used as templates for drug development projects.^4–7^

A central challenge in the synthesis of DRPs is the selective installation of disulfide bonds. Oxidative folding of these molecules often yields mixtures of disulfide isomers, particularly when the native fold is not strongly favored, or when the peptide sequence is appreciably divergent from natural templates.^2,8–10^ To overcome this, methods have been developed to direct oxidative folding pathways, including: orthogonal Cys protection strategies;^11–14^ preferential formation of Cys-Pen disulfides;^15–17^ and strategic placement of Pro residues to initiate peptide folding and promote local backbone conformations that favor correct disulfide pairing.^18,19^ Pro-mediated folding has also been used to improve the evolvability of DRPs for binder discovery.^20^

A range of proline analogues, with diverse physicochemical properties and conformational properties, have been reported.^21^ These are likely to have different effects on the folding properties of linear peptides. In this paper, we have investigated the use of a cyclic oxazolidine pseudoproline (ΨPro) as a conformationally biased, Pro-like motif. ΨPros are Ser-, Thr-, or Cys-derived cyclic structures originally developed by Mutter,^22–24^ which display kink-inducing effects that alter the local conformation of peptide backbones.^25,26^ Indeed, an early report showed that a ΨPro residue could direct the folding of the naturally occurring DRP sarafotoxin-S6b, with a 3:1 ratio of correct:incorrect disulfide bridging.^22^ Pseudoprolines provide potential to access more chemical diversity within disulfide-directing motifs: decoupling folding direction from Pro allows medicinal chemists to explore more chemical space in their DRP libraries without compromising on foldability. A few studies have demonstrated that incorporating ΨPro moieties in bioactive peptides can result in enhanced potency or modified target selectivity.^27–29^ Cys-derived ΨPro can also be used as masked Cys residues that are released at the site of action, for example in the design and synthesis of orally bioavailable prodrugs of ψ-GSH.^30^

To address this, we investigated the use of a cyclic oxazolidine pseudoproline (ΨPro) as a conformationally biased, Pro-like motif. ΨPros are Ser-, Thr-, or Cys-derived cyclic structures originally developed by Mutter in the 1990s.^22–24^ The ΨPro used in this study was a Thr-derived unsubstituted oxazolidine, Thr­(ψ^H,H^pro)-OH (compound **1**). Similarly to Pro, ΨPros display kink-inducing effects that alter the local conformation of peptide backbones.^25,26^

In this study, we demonstrate that a strategically placed pseudoproline, the Thr-derived unsubstituted oxazolidine, Thr­(ψ^H,H^pro)-OH **1**, matched Pro in its ability to direct the oxidative folding pathway of a model DRP. This represents an expansion in the chemical diversity of motifs that can impart oxidative folding preferences within DRP libraries.

We used cyclic ion mobility-mass spectrometry (cIM-MS) to investigate the conformational landscape of our DRP.^31^ Multipass cIM-MS experiments determined that our peptide displayed conformational heterogeneity rather than a single conformationally locked population. Conformer-selective collision activation (slice CA) experiments demonstrated that gas-phase interconversions between conformers took place.^32,33^ We attribute this conformational dynamism to *cis*/*trans* isomerization at the ΨPro amides. These observations provide evidence that conformational plasticity can persist even within multicyclic cystine-stabilized scaffolds. These experiments highlight the power of IM-MS in exploring the conformational landscapes and dynamics of DRPs, and how IM-MS experiments could provide information to better design in conformational constraints or adaptability, selectivity, or cell permeability.

## Experimental

### Procedures for Oxidative Folding

100 mM potassium phosphate monobasic buffer (pH = 7.4) containing 0.5 mM oxidized glutathione (GSSG) was added into a low-adsorption centrifuge tube, followed by the addition of linear peptide to a final concentration of 100 μM. After 4 h of reaction in a shaker at 37 °C, the reaction was quenched by 10% HPO_3_. Finally, the reaction was monitored by HPLC and the oxidation products were analyzed by mass spectrometry.

### Instrumentation and General Mass Spectrometry Procedures

MS experiments were conducted in positive mode on a SELECT SERIES Cyclic quadrupole time-of-flight mass spectrometer (cIM-qTOF) (Waters Corp., UK).^34^ The cIM-qTOF instrument was mass calibrated with 2 μg/μL NaI in 50/50 2-propanol/water over a range of 50–4000 *m/z*. Samples were directly infused using nano-ESI (nESI) gold-coated capillaries, produced in-house using a Flaming/Brown P-97 micropipette puller and a Quorum Q150RS sputter coater.

Source conditions for analysis were as follows: capillary 1.50 kV, sampling cone 60 V, source offset 20 V, source temperature 40 °C, cone gas 0 L/h, purge gas 0 L/h, and nanoflow gas 0.00 bar. The StepWave/ion guide region was operated with body gradient 25 V and head gradient 10 V, with ion guide 1 offset 3.0 V, ion guide 2 offset 0.3 V, and diffusion aperture 2 at 0.0 V. Ion guide traveling-wave parameters were 150 m/s (velocity) and 0.4 V (pulse height), with ion guide gas disabled.

Data were acquired in both MS mode (full scan) for native spectra and MS/MS mode for quadrupole isolation of specific charge states. Quadrupole/transfer optics were set to ion energy 1.5 V, prefilter 20.0 V, LM resolution 4.90, and HM resolution 15.00. The acquisition mass range was *m/z* 50–4000.

Cyclic IMS parameters were: dead time offset 1.50, entry offset 5.0 V, exit offset 2.0 V, gate traveling-wave velocity 375.0 m/s and gate traveling-wave height 6.0 V, with a global TW gradient of 2.5 and cyclic RF amplitude 250 V. Racetrack traveling-wave conditions were 375 m/s (velocity) with 24.0 V (TW height), 15.0 (TW start), and 35.0 (TW limit). The cyclic repeller was 100 V.

Associated upstream/downstream IM optics were: helium cell bias 35.0 V, helium DC entrance 30.0 V, helium DC exit 10.0 V; prearray bias 35.0 V and prearray gradient 5.0 V; postarray bias 5.0 V, postarray gradient 25.0 V, and postarray DC exit 2.0 V.

### Methods for Trypsin and GluC Digestion and Analysis

Disulfide bond connectivity in the major oxidative product (peptide **3**) was determined using a stepwise, diagnostic proteolysis workflow. A decision tree was applied in which the number of LC-MS-observed digest products generated by trypsin and GluC was used to distinguish the possible cystine pairings.

Trypsin digestion: to a 50 μL solution of isolated oxidative peptide (50 μg peptide, 50 mM ammonium bicarbonate) was added trypsin (Promega Sequencing Grade), to give a final trypsin:peptide ratio of 1:50 (w/w). The solution was vortexed and centrifuged. Sample was incubated at 37 °C for 3 h. Trypsin was quenched by the addition of 0.5% TFA. Prior to injection into the LC-MS, digest products were desalted using a ZipTip.

GluC digestion: to a 50 μL solution of isolated oxidative peptide (50 μg peptide, 50 mM ammonium bicarbonate) was added GluC (Promega Sequencing Grade), to give a final GluC:peptide ratio of 1:20 (w/w). The solution was vortexed and centrifuged. Sample was incubated at 37 °C for 20 h. GluC was quenched by the addition of 0.5% TFA. Prior to injection into the LC-MS, digest products were desalted using a ZipTip.

### cIM-MS Experimental

Multi-pass experiments were performed for peptide **3** on the cIM-qTOF instrument. The duration of sample injection was kept constant, while the separation time was varied to increase the number of times the ion packet traversed the cyclic IM device. During the inject step (10.00 ms), the prearray was operated at a gradient of 85.00 V and bias of 70.00 V, with an offset of 45.00 V and an array traveling-wave height of 2.00 V (forward). For the separate step, the prearray gradient (85.00 V) and bias (70.00 V) were maintained, while the offset was set to 70.00 V and the array traveling-wave height increased to 35.00 V (sideways); the separation duration was adjusted according to the number of passes, using 5.00 ms (1 pass), 12.00 ms (2 pass), 23.00 ms (3 pass), or 33.00 ms (4 pass). Finally, during eject and acquire, the prearray remained at a gradient of 85.00 V and bias of 70.00 V, with the offset returned to 45.00 V and the array traveling-wave height set to 25.00 V (forward).

### IM-CA-IM-MS Experimental

Conformers A and C were selected for IM-CA-IM-MS (slice-CA) experiments by using the ion mobility sequence parameters detailed in Table S3. Different ion populations were sliced out by varying the durations of the Eject, Eject to Pre Store, and Hold and Ejection functions in the cyclic ion mobility sequence. Sliced populations were activated upon reinjection by increasing the voltage of the prearray gradient. The prearray bias was fixed at 70 V during reinjection to give variable potential energy gradients. Increases of +10 V were applied to give both ion mobility and mass spectra of sliced ion populations that have been subjected to CA.

### Population Tracking Using Gaussian Functions

To determine the relative populations of conformers in IM-MS experiments, population tracking was performed by using Gaussian functions. Peak tops were manually selected from ion mobility spectra and peak centers were fixed. The height and width of each function were adjusted until the sum of all functions either converged with or had a low RMSD with data. This operation was performed manually using the OriginPro 2023b software. All resolved populations were included in the composite fit, and fit quality was assessed from the residual distribution, coefficient of determination (*R* ^2^), root-mean-square residual, and maximum absolute residual. The area under each fitted Gaussian was used as the measure of conformer abundance. For the reported α-versus-γ population analysis, the fitted intermediate β component remained included in the deconvolution but was excluded from the percentage denominator; α and γ were therefore normalized relative to *A*_α_ + *A*_γ_ and expressed as conditional percentages summing to 100% at each voltage.

## Results and Discussion

### Synthesis of Fmoc-Thr­(ψ^H,H^pro)-OH, **1**

To enable the synthesis of a model peptide with an oxazolidine moiety for the direction of oxidative folding toward a single isomer, we decided to synthesize and incorporate an Fmoc-Thr­(ψ^H,H^pro)-OH ΨPro moiety **1**. ΨPro building blocks have previously been developed as backbone protecting groups for Ser, Thr, or Cys residues in solid-phase peptide synthesis (SPPS) to prevent aggregation of difficult sequences on-resin during synthesis.^35^ These Ser/Thr-derived oxazolidine and Cys-derived thiazolidine ΨPro are known to nucleate peptide folding in a manner similar to Pro.^25,26^ This effect comes from a lowered energy barrier for *cis*/*trans* interconversions of the amide bond geometry.^36^ They are typically prepared *via* reaction of aldehydes or ketones with Ser/Thr/Cys. These ΨPro building blocks—Fmoc-Ser/Thr­(ψ^Me,Me^pro)-OH, Fmoc-Thr­(ψ^Me,Me^pro)-OH, and Fmoc-Cys­(ψ^H,Dmp^pro)-OH—were originally designed to be acid labile, so that they can be easily removed during global deprotection/resin cleavage with TFA at the end of the synthesis. A few are commercially available, however most of these ΨPro must be incorporated as dipeptides, as coupling yields are lowered by steric hindrance of the secondary amine by the dimethyl or Dmp groups.

For our purposes, however, we required an acid-stable ΨPro that would remain intact following peptide cleavage conditions (95% TFA). We synthesized the unsubstituted ΨPro **1** by reacting Fmoc-Thr-OH with paraformaldehyde in the presence of a catalytic quantity of (1*S*)-(+)-10-camphorsulfonic acid (Scheme 1, Figures S1-S4). Synthesis was achieved in reasonable yield, and purification was achieved using flash chromatography. This moiety has been used to try and direct disulfide bond formation in Sarafotoxin-S6b previously, though this attempt was unsuccessful.^22^

**Scheme 1:**
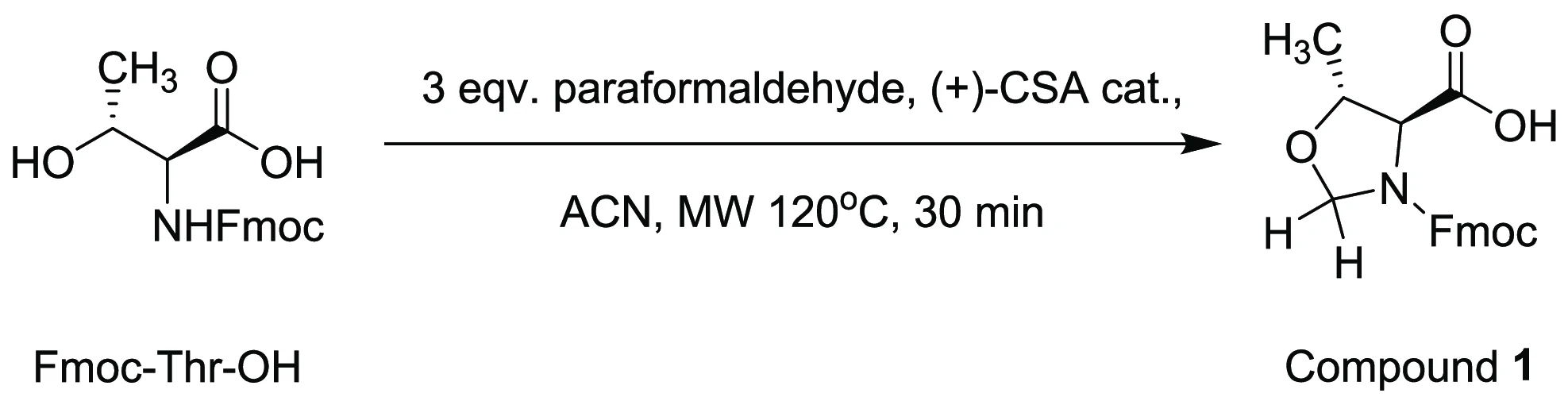
Synthesis of Compound **1**

To test the acid stability of compound **1**, the compound was solubilized in 10% and 50% TFA/H_2_O and analyzed with analytical HPLC at timepoints that are relevant to a peptide cleavage protocol (0–3 h). Across all timepoints, there was no apparent hydrolysis of compound **1** to form Fmoc-Thr-OH. No new peaks emerged in the analytical HPLC traces that could be attributed to the formation of Fmoc-Thr-OH, nor did the intensity of the peak associated with compound **1** reduce over time (Figure S5).

### Design and Synthesis of a Pseudoproline-Containing DRP

The selective and efficient formation of disulfide bonds remains a central challenge in the synthesis of disulfide-rich peptides (DRPs). One established strategy for directing oxidative folding is the incorporation of Pro within a turn-inducing motif to promote proximity and orientation of specific Cys residues. Among these, the CPXXC motif has been shown to be highly effective in promoting selective disulfide bond formation.^18^ The presence of Pro in this sequence induces a tight β-turn that preorganizes the peptide backbone, increasing the local concentration of paired Cys residues relative to one another and thereby directing the oxidative folding pathway toward a given disulfide isomer.

To explore whether this fold-directing effect could be retained while introducing greater chemical diversity to the motif, we designed a modified version of the CPIEC variant of the CPXXC motif, chosen for its highly directed oxidative folding, in which the Pro residue was substituted with compound **1**, a Thr-derived ΨPro.

Our design substituted the Pro in the CPXXC motif with a ΨPro, yielding a CΨPXXC motif embedded within an 18-residue peptide derived from a previously studied DRP scaffold.^18^ The hypothesis guiding this design was that compound **1**, like Pro, would induce a local conformational kink that promotes the productive alignment of Cys1 with Cys4, and Cys2 with Cys3, directing oxidative folding toward the antiparallel disulfide isomer.

We synthesized linear peptide **2** using solid-phase peptide synthesis (SPPS) methodology. General methods, specific protocols and characterization are outlined in full in the Supplementary Information (Tables S1, S2, Figures S6, S7). Standard SPPS was conducted on preloaded Fmoc-Gly-NovaSyn TGT resin to afford **2** (Scheme 2). Notably, compound **1** was incorporated as a single unit. Triple couplings with five equivalents of amino acid and coupling reagent were performed to ensure efficient couplings *N*-terminal to compound **1**. Resin cleavage and side-chain deprotection were carried out to give peptide **2** in 18% yield.

**Scheme 2:**
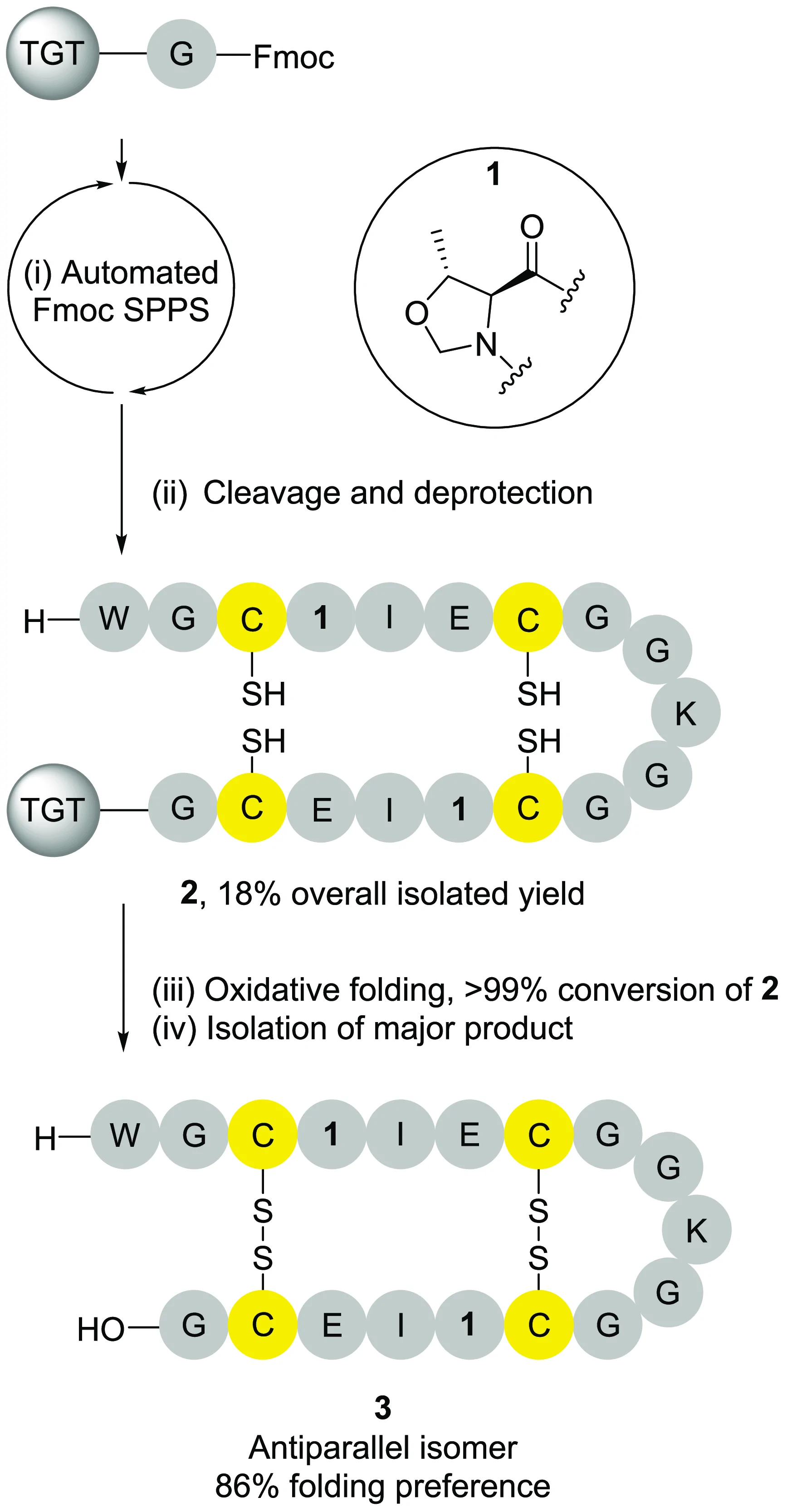
Synthesis of Peptide **3** [Fn sch2-fn1]

a
**Reagents and Conditions:** (i) Fmoc deprotection with 40%, then 20% piperidine in DMF, amino acid coupling Fmoc-aa-OH, HATU, DIPEA, DMF, performed in cycles until completion of the peptide sequence; (ii) TFA, TIPS, DODT, H_2_O; (iii) H_2_O, KH_2_PO_4_, GSSG, 37°C, 4 h.

Oxidative folding of peptide **2** was carried out under nonenzymatic redox-buffer conditions using oxidized glutathione (GSSG) in phosphate buffer, pH 7.4, 37 °C (Scheme 2). Under these conditions, GSSG functions as a disulfide exchange mediator, promoting thiol-disulfide shuffling and favoring thermodynamically preferred disulfide connectivities. Of the three possible fully oxidized disulfide isomers, only two were formed. The ratio of major:minor product was 86:14 (Figure S9). Further experiments were required to map the disulfide connectivity within the major oxidative product. Peptide **3** was isolated using semi-preparative HPLC, prior to disulfide mapping.

### Disulfide Mapping *via* Nonreducing Proteolysis and MS Analysis Identifies the Antiparallel Isomer

Peptide **3** was afforded from peptide **2** with 86% selectivity. This folding preference is the same as what was observed in the CPXXC motif peptide.^18^ In the CPXXC peptide, the major oxidative product was the antiparallel Cys­(I-IV)/Cys­(II-III) isomer. To determine whether the same architecture was present in peptide **3**, a digestion-based disulfide mapping approach was used. These experiments rely on the presence of enzymatic digest sites that provide differential products depending on which positional disulfide isomer is present. The presence and positioning of a Lys residue and the two Glu residues enable this strategic digest approach to be taken using trypsin and GluC.^37^ A decision tree can be constructed that allows unambiguous assignment of disulfide connectivity *via* MS analysis of two samples of peptide **3**: one that has undergone a tryptic digest, and another that has undergone a GluC digest (Figure 1).

Peptide **3** was shown to be the antiparallel Cys­(I-IV)/Cys­(II-III) isomer, similarly to the major oxidative product of the analogous CPXXC peptide (Figure 1). Two digested samples of peptide **3** were prepared, one was treated with trypsin and one was treated with GluC. The tryptic digest analysis allowed elimination of the adjacent Cys­(I-II)/Cys­(III-IV) isomer as a candidate for the identity of peptide **3**. Given the mapped trypsin cleavage site, the adjacent Cys­(I-II)/Cys­(III-IV) isomer would produce two digest products that are not linked *via* a disulfide bond. Instead, MS analysis of the tryptic digest product shows a single disulfide-linked species, consistent with either the parallel Cys­(I-III)/Cys­(II-IV) or antiparallel Cys­(I-IV)/Cys­(II-III) isomers. The GluC digest was informative in elucidating whether peptide **3** was the parallel or antiparallel isomer. For the parallel Cys­(I-III)/Cys­(II-IV) isomer, GluC digestion would produce a single digest product that has undergone two proteolytic events but that remains a single covalently intact entity through disulfide linkages. What was experimentally observed, however, were two digest products with masses that were consistent with the theoretical internal and flanking digest products of the antiparallel Cys­(I-IV)/Cys­(II-III) isomer.

**Figure 1:**
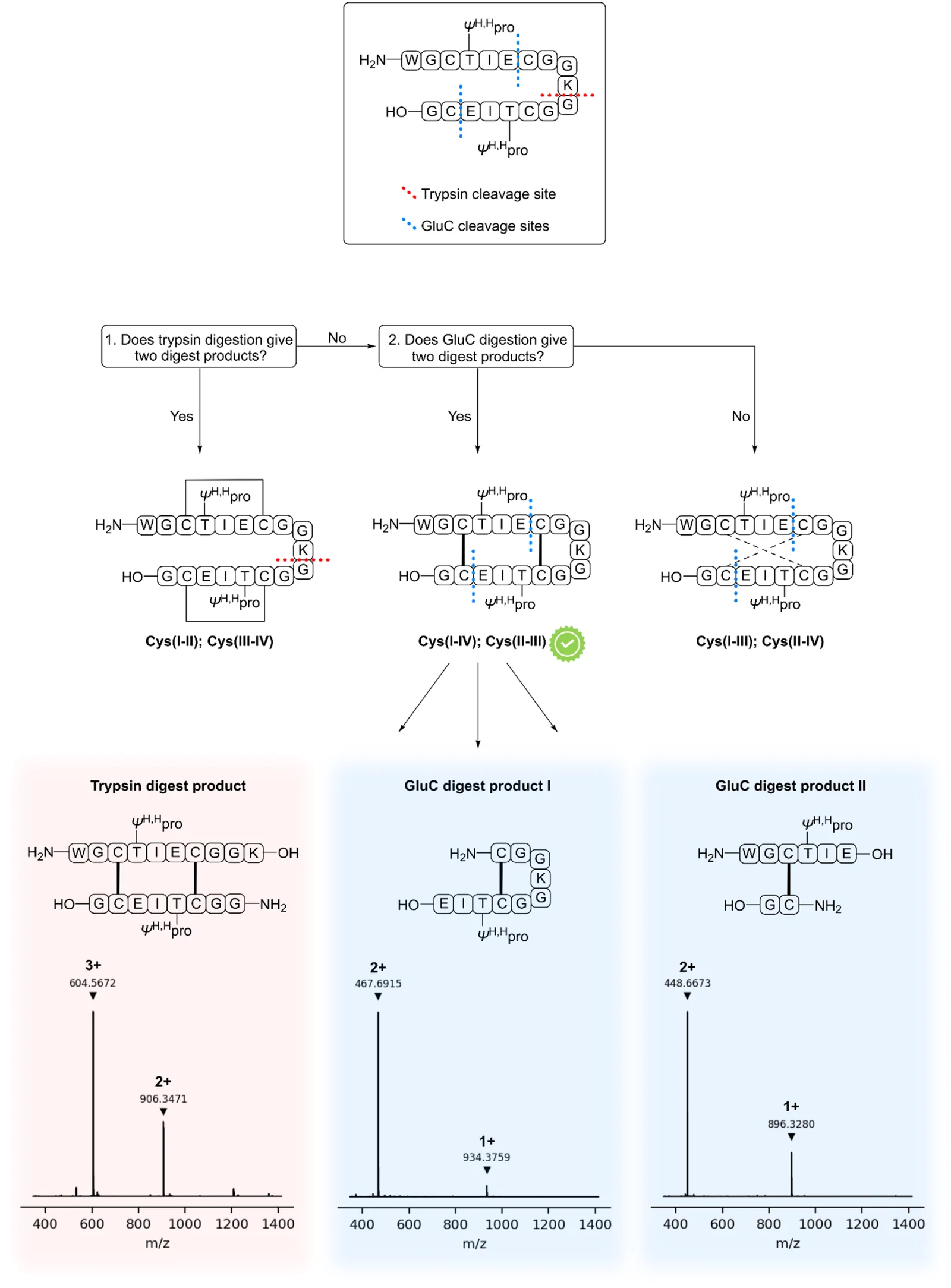
Decision tree for determination of the disulfide connectivity within peptide **3**. Each possible positional isomer from the complete oxidation of peptide **2** has a unique “digest fingerprint”, meaning sample digests using trypsin and GluC can be undertaken to definitively determine the disulfide bond connectivity within peptide **3**. Digest products from the treatment of peptide **3** with trypsin (red) and GluC (blue). Digestions yielded products that were consistent with peptide **3** being the antiparallel Cys­(I-IV)/Cys­(II-III) isomer.

### cIM-MS Shows that Peptide 3 Is Conformationally Heterogeneous Despite Its Disulfide Linkages

Conformational analyses of peptides are valuable because function is inherently linked to conformation. Peptide binding events depend on which conformers are present and their interconversion dynamics. Many peptides, especially DRPs, populate multiple energy basins. Knowing the number and size of these basins is important for peptide design. Understanding peptide conformational dynamics is crucial for guiding molecular design. For example, conformational compactness and rigidity are associated with proteolytic stability and target selectivity.^38,39^ Conversely, conformational heterogeneity can be associated with molecular chameleonicity and membrane permeability.^40^ The conformational dynamics of peptide **3** were explored using cyclic ion mobility-mass spectrometry (cIM-MS).

We used multipass cIM-MS to test whether peptide **3** is conformationally homogeneous or comprises multiple gas-phase conformers. The Waters SELECT Series cIM-qTOF instrument is uniquely well placed to explore this question.^34^ This cIM-qTOF has a cyclic mobility cell, around which ions can be sent a user-defined number of times. Increasing the mobility path length (multipass) enhances the resolving power to allow minor conformers to be separated from dominant species.^31,34,41^ These multipass cIM-MS experiments allow us to answer the question: are the two disulfide linkages sufficient to collapse or constrain peptide **3** to a single conformation, or are multiple conformers present?

The ion mobility spectra (Figure 2) show that peptide **3** exists as multiple conformers, demonstrating that the conformational constraint exerted by the disulfide linkages is not sufficient to provide an absolute conformational lock. As the number of passes was increased from one to four, the drift time of the 2+ conformers increased, and the two major conformers (α and γ, marked blue and green, respectively) were better resolved (Figure 2). After four passes of the cIM cell, the population marked in orange is subsumed by the two major conformers. This supports the notion that population B is not a “true” conformer, but is instead an intermediate that forms in the interconversions between major conformers α and γ. This behavior is analogous to the “bridge” reported by Eldrid et al. in cyclic IM-CA experiments, where signal between resolved conformers was attributed to dynamic interconversion within the conformational ensemble, rather than a fully discrete conformer.^32^

**Figure 2:**
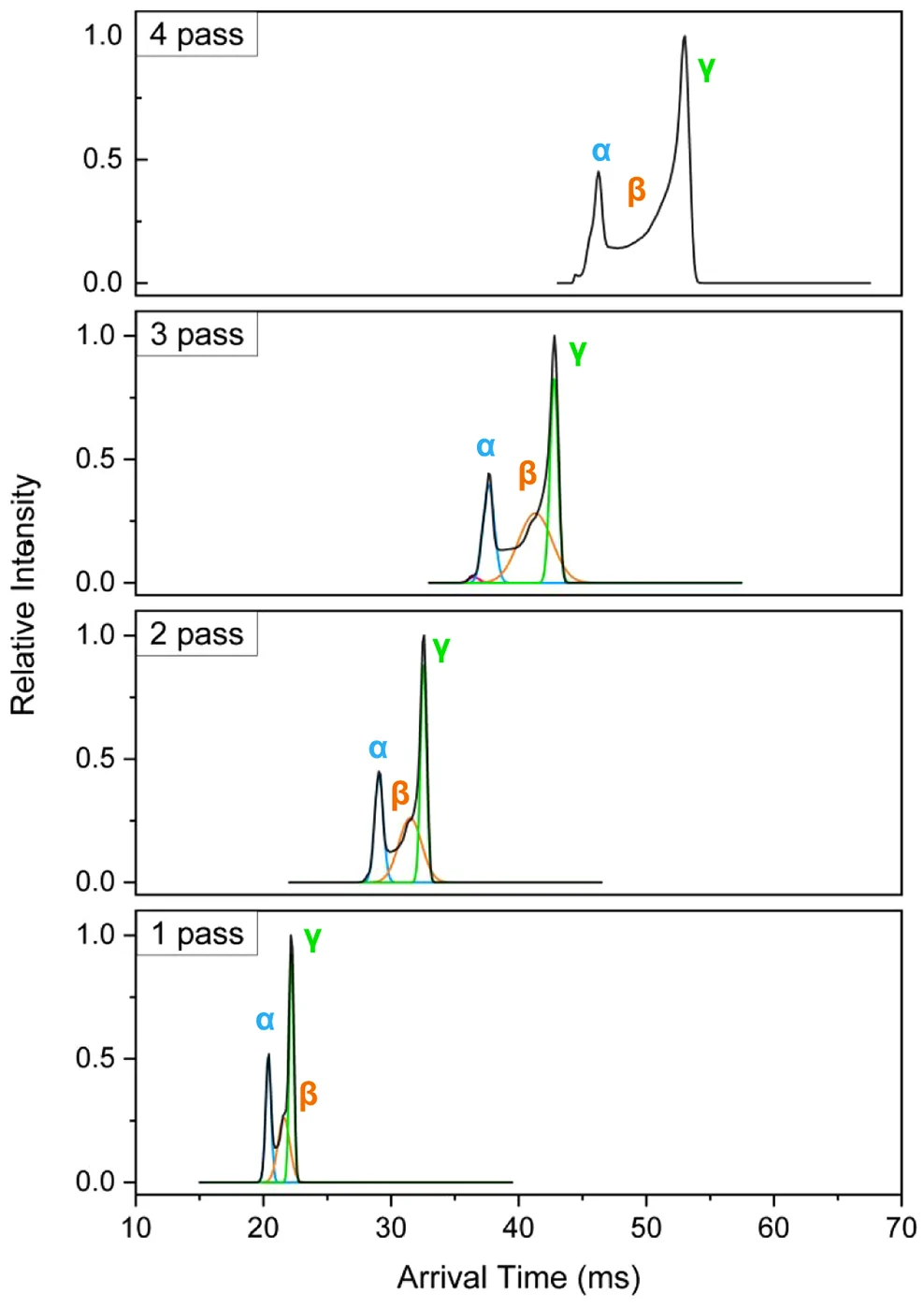
Ion mobility spectra from a multipass experiment on the 2+ ions of peptide **3**. Potential conformational families are marked with colored circles and are labeled α, β, or γ. Each additional pass around the cIM cell begins to resolve the major conformations α and γ (blue and green, respectively). Distinct conformations/intermediate states have had Gaussian peaks fitted with OriginPro, where possible. Family β (orange) can be considered a “bridge” intermediate/interconversion state between conformations α and γ.^32^

The cause of this conformational heterogeneity is likely to be the presence of the Thr­(ψ^H,H^pro) units in the peptide. These residues, similarly to Pro, have low energy barriers for interconversions of their amide geometry between *cis* and *trans*. Comparable observations have been made when using IM-MS to study the conformational landscape of bradykinin; experiments which substituted Pro residues for Ala collapsed the conformational ensemble to a single conformer.^42^ More generally, deconvolution of solution-phase NMR data and molecular dynamics simulations of similar cyclic β-turn hairpin peptides have established that these exist as ensembles of interconverting conformations, rather than being locked into a single defined secondary structure.^43–46^

### Mobility Selection Collision Activation Reveals Gas-Phase Interconversions between Conformers α and γ

To better understand the state-to-state interconversions of conformers α and γ, IM-CA-IM-MS experiments were conducted.^32^ In this workflow, the 2+ ions of peptide **3** enter the cIM device and undergo multipass separation before slicing of either conformer α and γ. In separate experiments, each conformer was then reinjected into the cIM device with increasing energies that result in higher collision activation and resulting conformational rearrangements. Rearranged conformers are then subject to further mobility separation before ejection to the ToF for detection.

Slicing was performed to produce ion packets in which a single conformer was largely overrepresented. Voltages between 0 and 50 V were applied to the sliced populations in 10 V increments (Figure 3, upper). The relative populations of conformers α and γ were determined using a Gaussian peak fitting function (Figure 3, lower). The application of modest external energy was sufficient to promote conversion between the two conformers. Upon incremental activation (10 to 50 V), each isolated population undergoes a conformational interconversion toward a common α:γ ratio of 1:1. Interconversions are bidirectional: α decreases while γ increases when α is sliced, and conversely γ decreases while α increases when γ is sliced. Bidirectionality here shows that α and γ are discrete minima on a similar energy landscape, separated by an energy barrier that is easily surmountable in the context of these IM-CA-IM-MS experiments. Practically, this means that the conformational heterogeneity of peptide **3** arises from a defined conformational rearrangement rather than a progressive structural extension. It is worth noting that the typical collision-induced unfolding (CIU) signature is conformational extension with additional energy.^32,47,48^ The conversion of conformer γ back to α under additional energy is counter to this CIU norm, supporting the notion of a specific barrier-crossing isomerization rather than generic unfolding.

**Figure 3:**
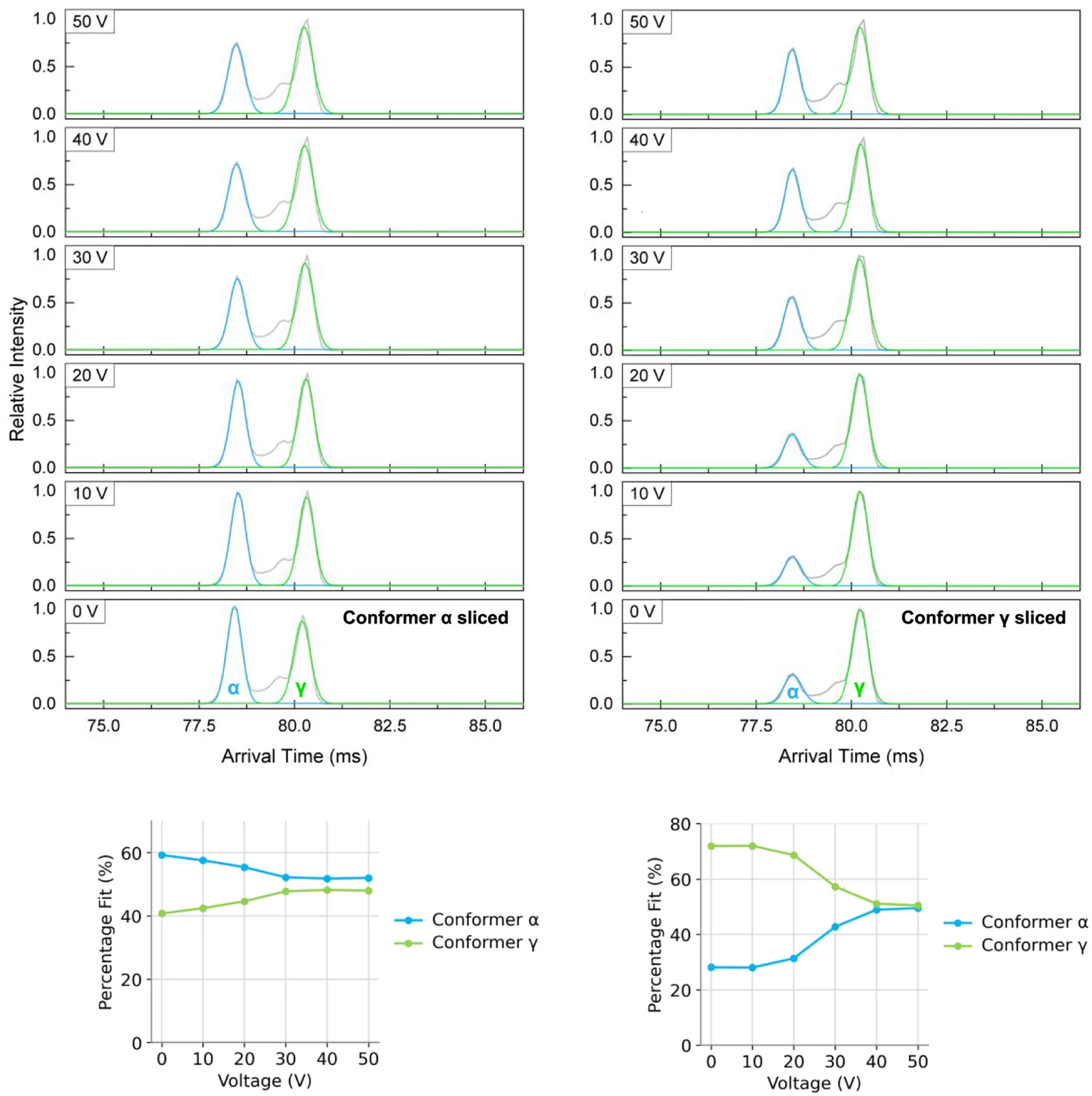
Mobility selection and activation of conformers α and γ of peptide **3**. Conformers were ejected to the prestore before being reinjected into the cIM cell with stepped voltage activations. Population tracking across different voltage activations reveals that an equilibrium of 1:1 was produced, suggesting gas-phase conformational interconversions.

We interpret the bidirectional exchange between α and γ as a ΨPro-anchored rearrangement. There appears to be a low-barrier-limited *cis*/*trans* X-ΨPro isomerization that toggles the local turn geometry and drives a reorganization of the peptide backbone within the disulfide scaffold.

## Conclusion

We set out to decouple Pro-dependent folding control from sequence constraints within a DRP scaffold. To achieve this, we prepared an acid-stable Thr-derived pseudoproline (compound **1**), verified its stability under peptide cleavage conditions, and installed it into a CΨPXXC motif within peptide **2**. Under mild, nonenzymatic redox conditions (GSSG, pH 7.4, 37 °C) peptide **2** was folded selectively to two of the three possible disulfide isomers with an 86:14 major:minor ratio. Nonreducing proteolysis with an MS readout unambiguously identified the major product, peptide **3**, as the antiparallel Cys­(I-IV)/Cys­(II-III) isomer.

cIM-MS revealed that, despite its multicyclic cystine scaffold, the oxidized peptide populates multiple gas-phase conformers. Slice CA experiments demonstrate bidirectional α↔γ interconversion under modest CA. Notably, the γ to α conversion upon additional energy runs counter to the usual CIU trend toward conformational extension, providing evidence for conformational isomerization rather than progressive unfolding. Together, these data indicate that the same ΨPro-anchored *cis*/*trans* amide isomerization that biases folding selectivity in solution most likely also underlies the conformational heterogeneity that is observed for peptide **3** in the gas phase.

Conceptually, this work expands the chemical palette for directing DRP folding beyond Pro while preserving fold preference and establishes an IM-CA-IM-MS workflow as a practical tool for the quantification of conformer populations and interchanges. Whether substitution of Pro for ΨPro within bioactive DRPs preserves target binding remains to be established, and matched binding and functional studies will be important in determining its utility for medicinal chemistry optimization. Future studies could also intentionally design conformational heterogeneity into or out of these scaffolds. For example, *cis*-biasing Pro analogues such as 4-(*S*)-fluoroproline could maintain directed oxidative folding while reducing heterogeneity within the oxidized DRP ensemble.^49^ Relating these gas-phase observations to solution-phase measurements and MD simulations would also be valuable.^50^ This work contributes to expanding the chemical motifs available for controlling both the oxidative folding of DRPs and their conformational landscapes.

## Supplementary Material



## Notes

General experimental, including details on the synthesis and analysis of compound 1; general peptide experimental information; general peptide synthesis methods; further information for IM-CA-IM-MS experiments; and details on Gaussian peak fitting residuals (PDF)

## Abbreviations Used

CA: collision activation
CIU: collision-induced unfolding
cIM-MS: cyclic ion mobility-mass spectrometry
cIM-qTOF: cyclic quadrupole time-of-flight mass spectrometer
DC: direct current
DRP: disulfide-rich peptide
HM resolution: high-mass resolution
HPLC: high-performance liquid chromatography
ICK: inhibitory cystine knot
IM: ion mobility
IM-CA-IM-MS: ion mobility-collision activation-ion mobility-mass spectrometry
IM-MS: ion mobility-mass spectrometry
IM^n^: stacked ion mobility
LC-MS: liquid chromatography-mass spectrometry
LM resolution: low-mass resolution
MD: molecular dynamics
MS: mass spectrometry
MW: microwave
pDRE: programmable Dynamic Range Enhancement
RF: radio frequency
slice CA: slice collision activation
SPPS: solid-phase peptide synthesis
TW: traveling wave
ΨPro: pseudoproline
