# Supplementary material for: Directed Oxidative Folding of Disulfide-Rich Peptides (DRPs) Using a Pseudoproline Unit: Ion Mobility-Mass Spectrometry (IM-MS) Reveals the Conformational Landscape of the DRP
Source: Anal Chem. 2026 Sep 17;98(38):28037–45. doi: 10.1021/acs.analchem.6c02816 (PMC13629162; doi:10.1021/acs.analchem.6c02816)
Supplement: Supplementary file 1 [file ac6c02816_si_001.pdf]

Supporting Information: Directed oxidative folding of disulfide-rich peptides (DRP) using a pseudoproline unit: ion mobility-mass spectrometry (IM-MS) reveals the conformational landscape of the DRP

Declan T Cook[a], Kish R Adoni[b], Konstantinos Thalassinos*[b,c], and Alethea B Tabor*[a]

[a] Dr Declan T Cook, Professor Alethea B Tabor, Department of Chemistry, University College London, 20 Gordon Street, London WC1H 0AJ, United Kingdom.
E-mail: a.b.tabor@ucl.ac.uk

[b] Dr Kish R Adoni, Professor Konstantinos Thalassinos, Department of Structural and Molecular Biology, University College London, Division of Biosciences, University College London WC1E 6BT, United Kingdom.
Email: k.thalassinos@ucl.ac.uk

[c] Professor Konstantinos Thalassinos, Institute of Structural and Molecular Biology, Birkbeck College, School of Natural Sciences, University of London, London WC1E 7HX, United Kingdom

Professors Tabor and Thalassinos are both corresponding authors for this paper.

Table of Contents

General Experimental **2**

Synthesis of Fmoc-Thr($\Psi^{H,H}$pro)-OH, compound 1 .... **2**

__Figure S1.__ ^{1}H NMR of compound __1__ (500 MHz, $CDCl_3$) *3*

__Figure S2.__ ^{13}C NMR of compound __1__ (126 MHz, $CDCl_3$) *4*

__Figure S3.__ QC MS analysis of compound __1__ *4*

__Figure S4.__ QC analytical HPLC of compound __1__ *5*

__Figure S5__. Acid stability of __1__ *5*

General peptide experimental **6**

__Table S1.__ Side chain protecting groups used on N-α-Fmoc-protected amino acids in solid-phase peptide synthesis. *6*

General peptide synthesis methods **7**

Peptide synthesis **8**

Synthesis of linear peptide 2 .... **8**

__Table S2.__ Quantities of protected amino acids used for the synthesis of peptide 2. .... *8*

__Figure S6.__ QC MS of peptide __2__ *9*

__Figure S7.__ QC analytical HPLC of peptide __2__ *9*

Synthesis of oxidised peptide 3 .... **10**

__Figure S8.__ QC MS of isolated peptide __3__. *10*

__Figure S9.__ Analytical HPLC traces to show the oxidation profile of peptide __2__ and isolation of its major oxidative product, peptide __3__. *11*

Further information for IM-CA-IM-MS experiments **12**

__Table S3__. Detailed cyclic sequence for slice-CA experiments conducted on peptide __3__. *12*

Gaussian peak fitting residuals *13*

Figure S10. Residual analysis of Gaussian deconvolution across collision-voltage conditions. *14*

General Experimental

All reagents obtained from chemical suppliers were used without prior purification. All solvents were obtained from Fluorochem, Sigma-Aldrich, VWR, Acros Organics or Fisher Scientific. Brine refers to a saturated solution of sodium chloride in water. Water was distilled using an Elga Purelab Option R7 water purifier. TLC analysis was on aluminium backed Sigma-Aldrich TLC places with F254 fluorescent indicator. Flash column chromatography was carried out using Merck silica gel 60 (40-60 μm).

^{1}H and ^{13}C spectra were recorded for the assignment of compounds on a Bruker Avance NEO 500. Chemical shifts (δ) are given in ppm relative to the solvent signal and coupling constants (*J*) are given in Hz. Abbreviations used in ^{1}H NMR assignments are as follows: Ar = aromatic, appt = apparent, br = broad, s = singlet, d = doublet, t = triplet, m = multiplet, dd = doublet of doublets, ddd = doublet of doublets of doublets, dddd = doublet of doublets of doublets of doublets, dt = doublet of triplets, ddt = doublet of doublet of triplets, tdd = triplet of doublets of doublets. Data processing was carried out using MestReNova Edition, version 14.1.2 (Mestrelab Research S.L.).

Synthesis of Fmoc-Thr($\Psi^{H,H}$pro)-OH, compound 1

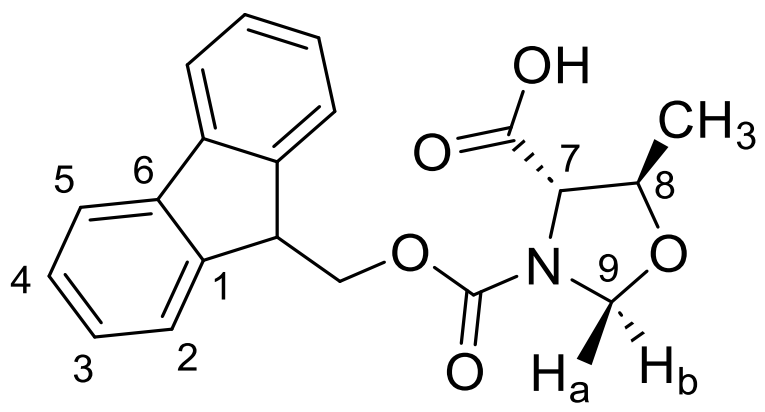

To Fmoc-Thr-OH (3 mmol, 1024 mg) in a microwave reactor tube with a magnetic stirrer, was added solid paraformaldehyde (12 mmol, 360 mg), CSA (0.26 mmol, 60 mg, 8.7mol%) and acetonitrile (15 mL). The reactor tube was then microwaved at 120ºC for 30 mins. The resultant cooled mixture was concentrated *in vacuo,* then loaded onto a silica column for flash chromatography. Purification by flash chromatography was achieved using MeOH/DCM (1/99) spiked with 0.1% acetic acid as eluent. Product was concentrated *in vacuo* to give a white powder. Lyophilisation from water/ACN (1:1) yields a sticky powder (380 mg, 36%).

R_f 0.40 (MeOH:DCM, 1:9)

δ_H (500 MHz, 60ºC, Chloroform-*d*) 7.75 (2 H, d, *J* 7.5, Ar*H*(5)), 7.55 (2 H, dd, *J* 7.5, 3.2, Ar*H*(2)), 7.39 (2 H, t, *J* 7.6, Ar*H*(4)), 7.31 (2 H, t, *J* 7.5, Ar*H*(3)), 5.13 (1 H, br d, H_b), 4.80 (1 H, d, *J* 4.3, *H*(7)), 4.51 (2 H, d, *J* 6.1, FmocCHCH_2), 4.29 (1 H, br d, H_a), 4.23 (1 H, t, *J* 6.1, FmocC*H*CH$_2$), 3.94 (1 H, br m, *H*(8)), 1.45 (3 H, d, *J* 6.1, CH_3).

δ_C (126 MHz, CDCl$_3$) 154.09 (CO_2H), 143.67 (*C*6), 141.47 (*C*1), 127.98 (*C*4), 127.28 (*C*3), 125.34, 125.01 (*C*2), 120.20 (*C*5), 78.72 (*C*9), 78.49 (*C*7), 68.31 (FmocCH*C*H$_2$), 63.38 (*C*8), 47.20 (Fmoc*C*HCH$_2$), 18.63 (*C*H$_3$). Fmoc carbamate carbonyl carbon signals not visible, likely due to peak broadening as a result of slow exchanging conformers.

Mass Analysis: product has an elemental composition of $C_{20}H_{19}NO_5$. Calculated exact mass of product is 353.12632 Da.

LC-MS: *m/z* calculated for $C_{20}H_{20}NO_5$ ([M+H]$^+$) 354.1341, LC-MS analysis observed 354.2807; *m/z* calculated for $C_{40}H_{39}N_2O_{10}$ ([2M+H]$^+$) 707.2599, LC-MS analysis observed 707.6024. Isotopic spacing of the $C_{20}H_{20}NO_5$ ([M+H]$^+$) ion shows definitively that this species is singly charged.

Analytical HPLC trace: Rt = 21.72 mins (5-95% B over 30 mins, λ = 214 nm).

Figure S1. ^{1}H NMR of compound **1** (500 MHz, $CDCl_3$)

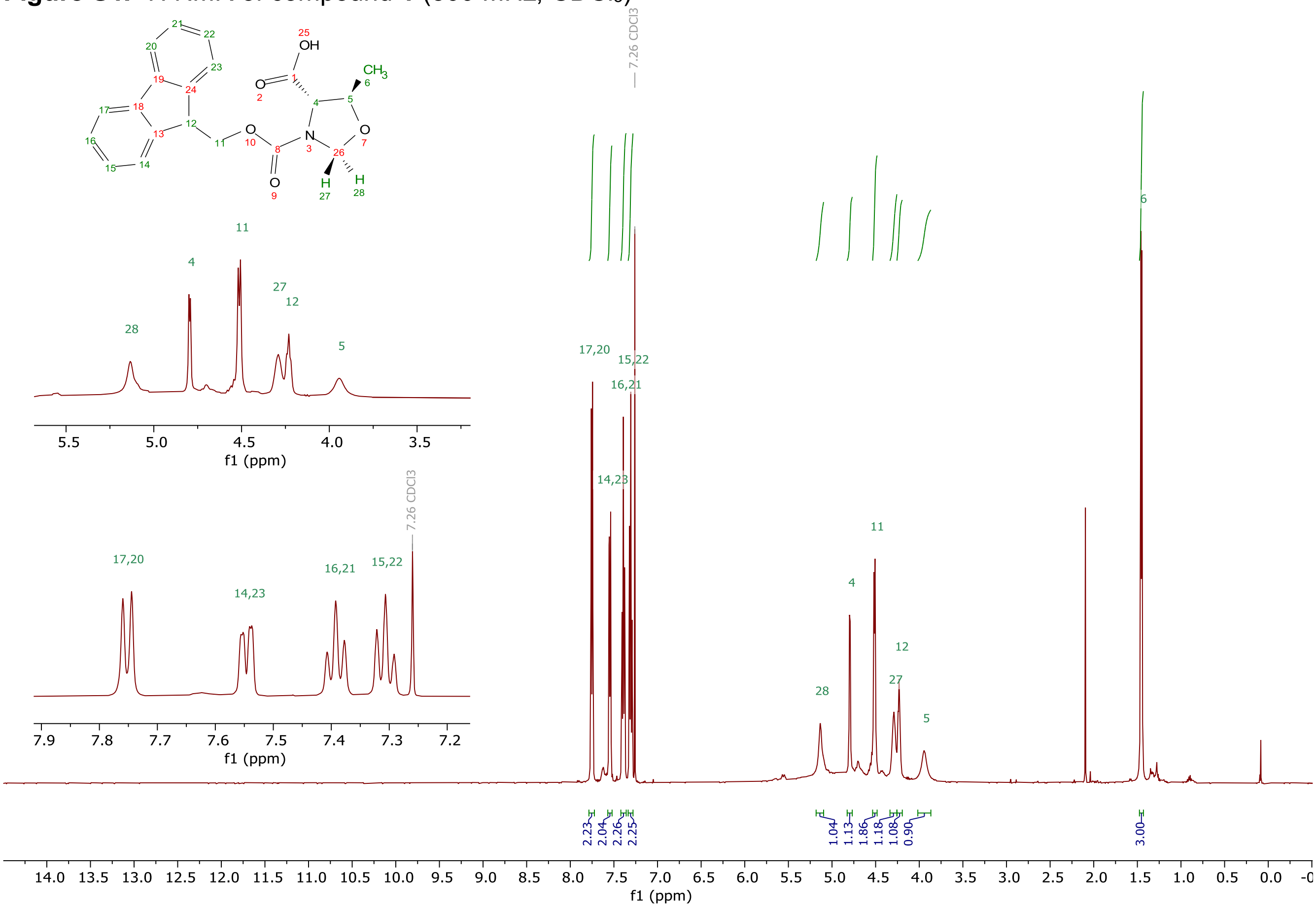

Figure S2. ^{13}C NMR of compound **1** (126 MHz, $CDCl_3$)

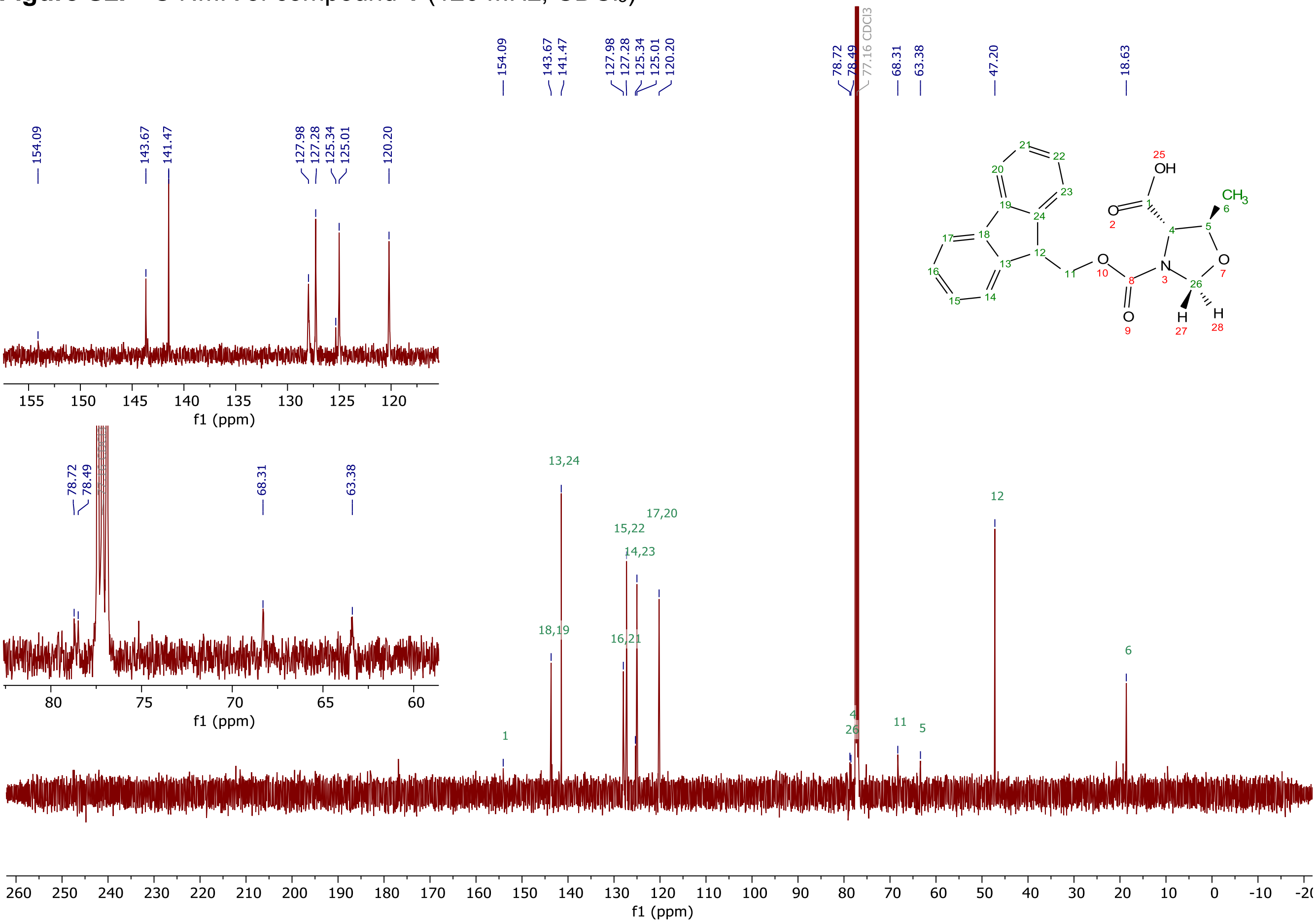

Figure S3. QC MS analysis of compound **1**

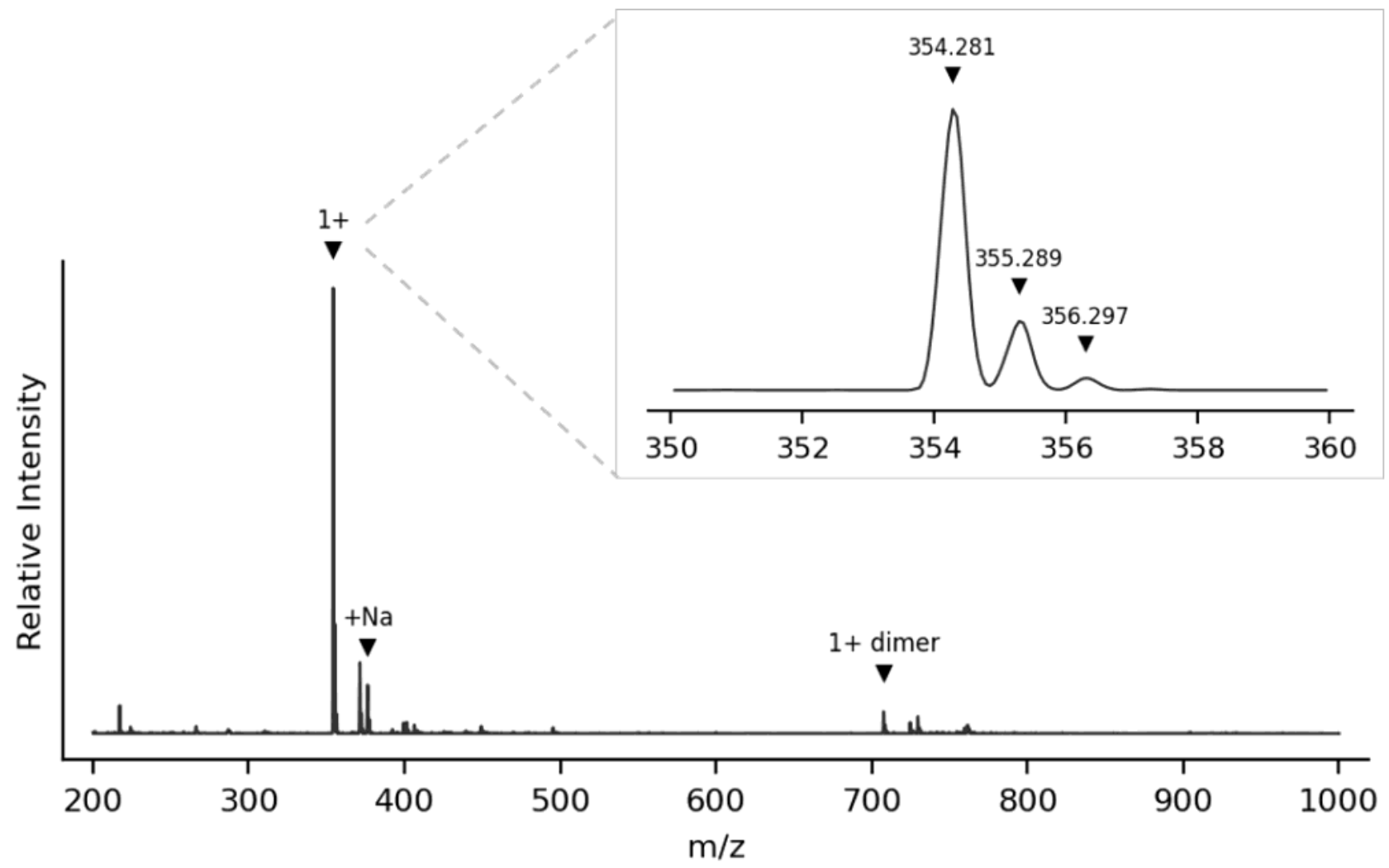

Figure S4. QC analytical HPLC of compound **1**

(5-95% B over 30 mins, flow rate of 1 mL min^{-1}, λ = 214 nm). Retention time 21.72 min.

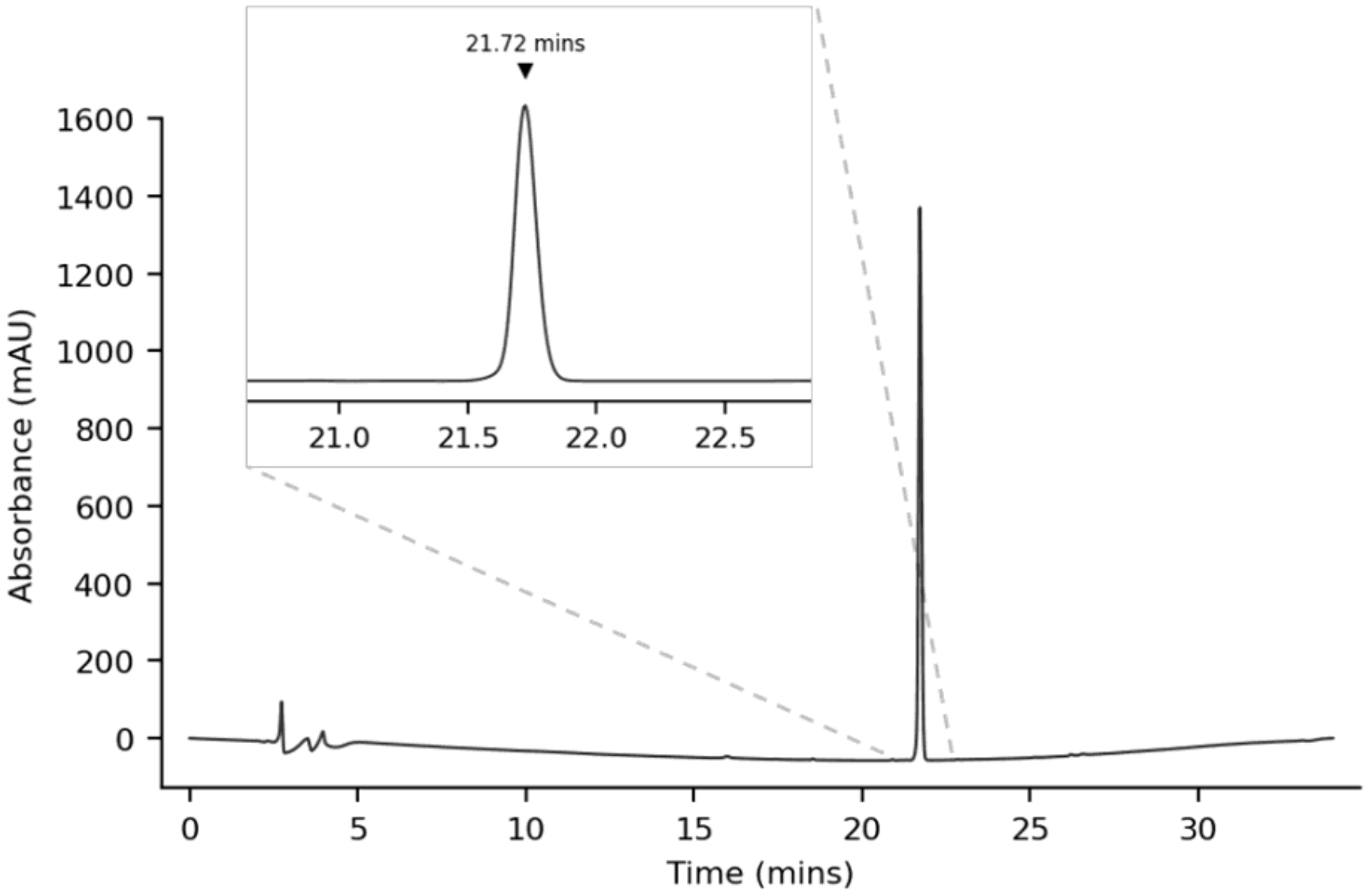

Figure S5. Acid stability of **1**

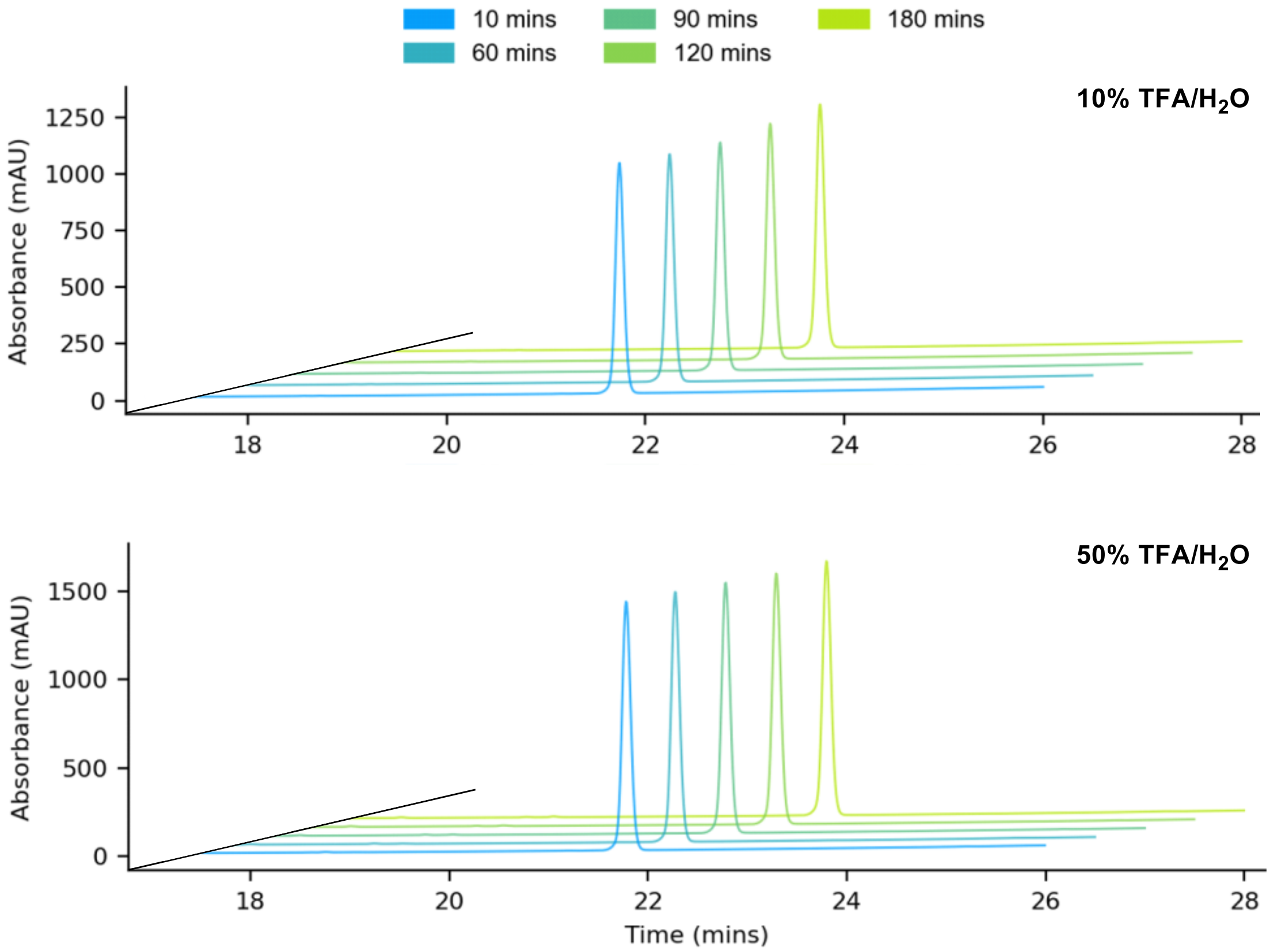

General peptide experimental

All peptides were synthesised using an Fmoc protecting group strategy. Protected amino acids were purchased from either Novabiochem or Fluorochem. HATU was purchased from Fluorochem. TIPS was purchased from Thermo Fischer Scientific. All remaining coupling reagents, bases, and solvents were purchased from Sigma-Aldrich. Protected amino acids and coupling reagents were stored at -20°C. HPLC-grade water, DMF, acetonitrile, and NMP were used throughout peptide syntheses and purifications. Side chain protecting groups were as described in **Table S1**.

Peptides were dried by flash-freezing in liquid nitrogen followed by lyophilisation using a VirTis BenchTop Pro (SP Scientific, Warminster, USA).

Amino Acid	Side chain protecting group
Cysteine	Trt
Glutamic acid	*t*-Bu
Glycine	-
Isoleucine	-
Lysine	Boc
Tryptophan	Boc

Table S1. Side chain protecting groups used on N-α-Fmoc-protected amino acids in solid-phase peptide synthesis.

Peptides were synthesised using a Syro I Fully Automated Parallel Peptide Synthesiser. All steps were performed at room temperature in a 5 mL polypropylene reaction syringe with a frit. The resin was washed with copious DMF following coupling and deprotection steps. Washing refers to the addition of solvent followed by immediate evacuation under vacuum. Following the cleavage from resins, an LC-MS of the crude peptide was acquired to confirm the success of the synthesis before purification.

Peptides were purified by semi-preparative reverse phase HPLC on an Agilent 1260 Infinity LC System with a G7165A – 1260 Infinity II Multiple Wavelength Detector, G7161A – 1260 Infinity II Binary Pump and a G7157A – 1260 Infinity II Preparative Autosampler. Chromatograms were analysed using OpenLab ChemStation (Agilent). A Phenomenex Jupiter Proteo 4 µm C12 250 x 10.0 mm column was used for purification with detection at 214, 230 and/or 254 nm. Quality-check (QC) analytical HPLC of purified peptides were performed on an Agilent Technologies 1260 Infinity system using an Avantor ACE 5 µm C18-AR 150 x 4.6 mm column. Water (0.1% TFA) and acetonitrile (0.1% TFA) were used as solvent A and B, respectively. For QC analytical HPLC of purified peptides, a 33-minute HPLC run was used; 5% solvent B for 3 minutes followed by a 5-95% solvent B gradient over 30 minutes at a flow rate of 1 mL min^{-1} with detection at 214 nm. Liquid chromatography-mass spectrometry (LCMS) spectra were recorded on a Waters Acquity UPLC SQD using a linear gradient of 5 - 95% B over 5 min (A = water, B = acetonitrile, 0.1% formic acid) with a C8 column, flow rate 0.6 mL min^{-1}. Analysis of the chromatograms was conducted using MassLynx software.

General peptide synthesis methods

Automated SPPS using the Syro synthesiser was conducted within 5 mL reaction syringes with a frit. Total reagent volumes were fixed at 1.5 mL, with reagent quantities and concentrations calculated from the quantity and loading of the resin. Solvent was evacuated by vacuum pump over 30 sec. Fmoc deprotection steps were carried out using 40% or 20% piperidine in DMF (1.5 mL) with intermittent shaking of 20 sec on and 40 sec off. Elongation steps were conducted by the sequential addition of Fmoc-protected amino acid in DMF (0.6 mL, 5 eq), HATU in DMF (0.6 mL, 5 eq) and DIPEA in NMP (0.3 mL, 10 eq) with intermittent shaking of 30 sec on and 60 sec off for 40 min. The coupling was repeated to ensure complete coupling.
The following protocols were followed for automated SPPS:

1. Resin swelling
   a. DMF (2.5 mL) was added
   b. Wait 20 min
   c. Evacuate
2. Fmoc deprotection
   a. 40% piperidine in DMF added (1.5 mL)
   b. Interval shaking for 3 min
   c. Evacuate
   d. 20% piperidine in DMF added (1.5 mL)
   e. Interval shaking for 10 min
   f. Evacuate
   g. Wash with DMF (6 x 1.5 mL)
3. Amino acid coupling
   a. Fmoc amino acid in DMF added (0.6 mL, 5 eq)
   b. HATU in DMF added (0.6 mL, 5 eq)
   c. DIPEA in NMP added (0.3 mL, 10 eq)
   d. Interval shaking for 40 min
   e. Evacuate
   f. Wash with DMF (2 x 1.5 mL)
   g. Repeat 3a – 3e
   h. Wash with DMF (4 x 1.5 mL)
4. Repeat steps 2 – 3
5. Fmoc deprotection

After the final Fmoc deprotection and washing of peptides synthesised on the Syro, the protocol for the cleavage and work-up of the peptide from the resin was as follows:

Cleavage and work-up

Cleavage cocktail for cysteine-containing peptides:
TFA:TIPS: 2,2′-(ethylenedioxy)diethanethiol (DODT):H2O (92.5:2.5:2.5:2.5)
The resin was shrunk by washing sequentially with CH_2Cl_2 (3 x 3 mL), MeOH (3 x 3 mL) and diethyl ether (3 x 3 mL). Cleavage cocktail (3 mL) was added to the resin and agitated for 3 h. The cocktail was evacuated directly into ice-cold diethyl ether (30 mL) in a 50 mL Falcon tube forming a cloudy suspension. Further cleavage cocktail (1 mL) was added to the resin for 5 min before evacuation into the ice-cold diethyl ether. The suspension was then centrifuged at 4000 rpm for 10 min at 4°C. The supernatant was decanted, and the pellet re-suspended in ice-cold diethyl ether (30 mL). This was repeated for a total of three times, after which the peptide was dissolved in the minimum amount of water and lyophilised to give the crude peptide powder.

Peptide synthesis

Synthesis of linear peptide 2

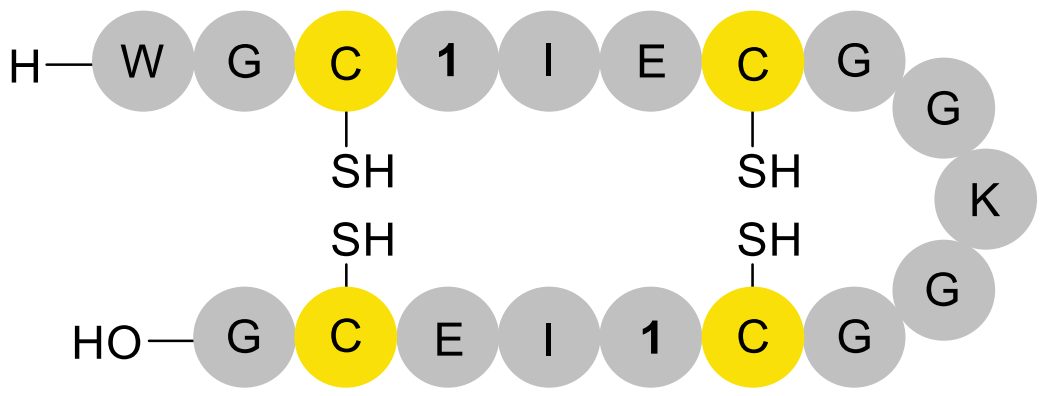

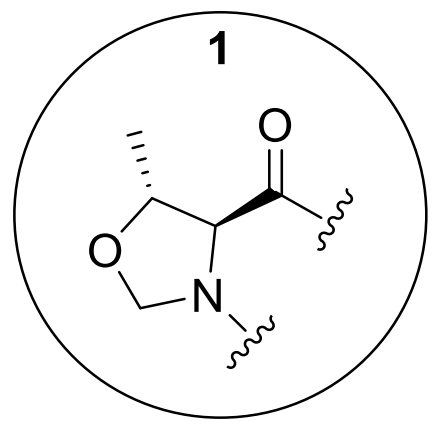

2

This peptide acid was synthesised on a 0.0315 mmol scale, using Fmoc-Gly-NovaSyn® TGT resin (resin loading 0.21 mmol/g, 300 mg of resin used). Automated SPPS was performed according to **General peptide synthesis methods**. Synthesis was carried out using the quantities of amino acids listed in **Table S2** below:

Amino Acid	Amino Acid Mass (mg)	Volume DMF (mL)
Fmoc-Cys(Trt)-OH	1175	6.77
Fmoc-Glu(O^{t}Bu)-OH	438	3.59
Fmoc-Gly-OH	741	9.00
Fmoc-Ile-OH	364	3.65
Fmoc-Lys(Boc)-OH	253	1.85
Compound **1**<br>*Fmoc-Thr(*$\Psi^{H,H}$*pro)-OH*	364	3.84
Fmoc-Trp(Boc)-OH	285	1.84

Table S2. Quantities of protected amino acids used for the synthesis of peptide 2.

Crude peptide was purified by semi-preparative RP-HPLC, using a Jupiter Proteo C12 (4 µm, 10.0 x 250 mm) column equipped with a 10 mm guard column. The gradient for purification was 5-34% Buffer B over 30 minutes, 5 mL/min. After lyophilisation, the peptide product was acquired as a fluffy white powder (10.2 mg, 18.0%) Peptide product retention time was 27.43 mins.

Mass Analysis: product has an elemental composition of $C_{73}H_{112}N_{20}O_{25}S_4$. Calculated exact mass of product is 1796.6990 Da.

Low-resolution LC-MS (ES-): *m/z* calculated for $C_{73}H_{111}N_{20}O_{25}S_4$ ($[M-H]^-$) 1795.69, LC-MS analysis observed 1795.66; *m/z* calculated for $C_{73}H_{110}N_{20}O_{25}S_4$ ($[M-2H]^{2-}$) 897.34, LC-MS analysis observed 897.39.

HRMS: *m/z* calculated for $C_{73}H_{113}N_{20}O_{25}S_4$ ($[M+H]^+$) 1797.7069, LC-MS analysis observed 1797.7070; *m/z* calculated for $C_{73}H_{114}N_{20}O_{25}S_4$ ($[M+2H]^{2+}$) 899.3573, LC-MS analysis observed 899.3572. $C_{73}H_{113}N_{20}O_{25}S_4Na$ ($[M+H+Na]^{2+}$) and $C_{73}H_{113}N_{20}O_{25}S_4K$ ($[M+H+K]^{2+}$) species are also observed at 910.3461 and 918.3293, respectively. Isotopic spacing of the $C_{73}H_{114}N_{20}O_{25}S_4$ ($[M+2H]^{2+}$) ion shows definitively that this species is doubly charged.

Estimated $\varepsilon_{(280\ nm)}$ 5500 M^{-1} cm^{-1} (based on the assumption that the molar extinction coefficient of this species, with the unnatural Thr($\Psi^{H,H}$pro) residues, is the same as the linear PIE peptide), mass of peptide = 10.2 mg.

Analytical HPLC trace: Rt = 12.67 mins (5-95% B over 30 mins, λ = 214 nm).

Figure S6. QC MS of peptide **2**

Inset is a zoomed region of the spectrum that shows the 2+ charge state (full annotation and characterisation listed above).

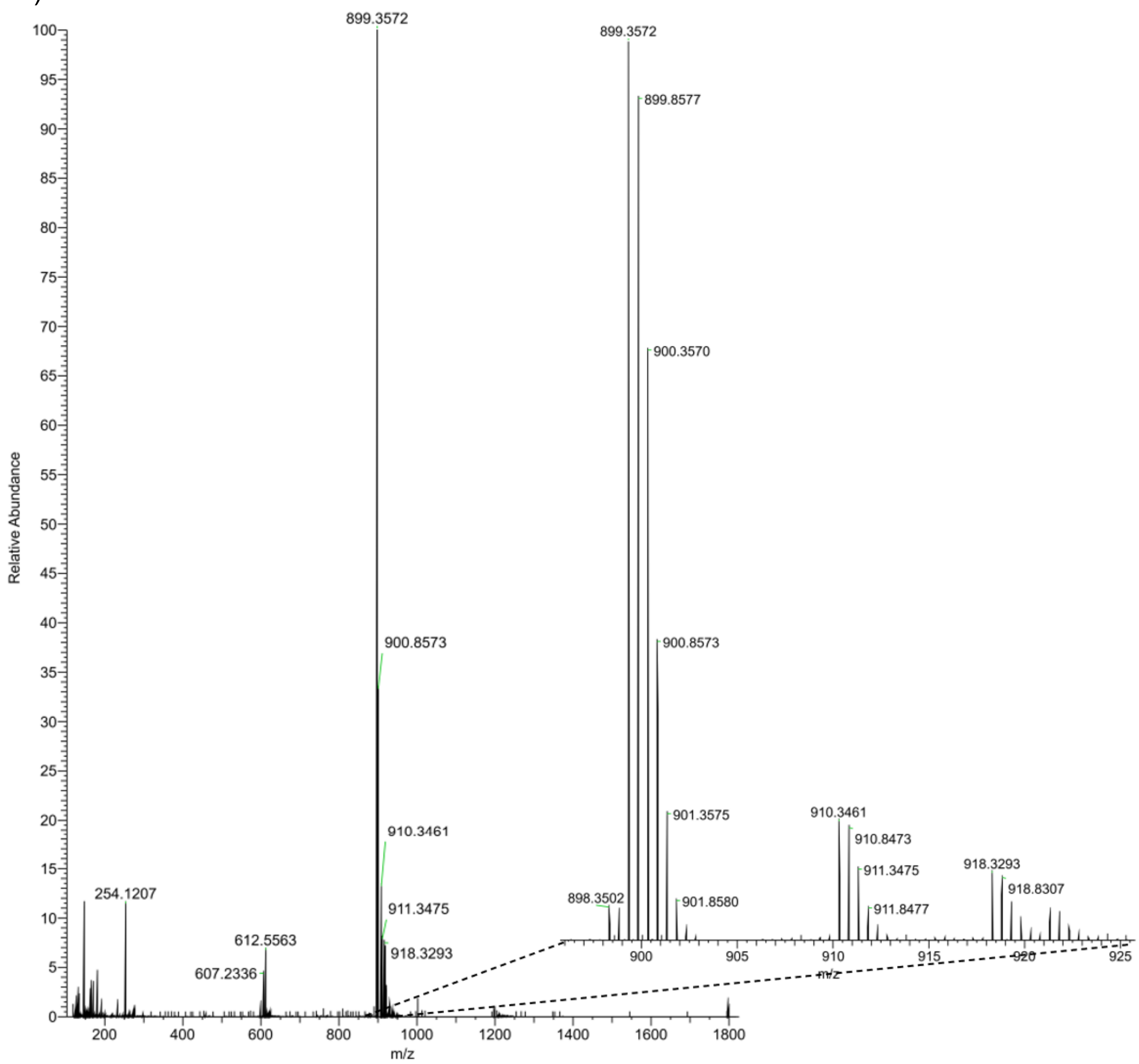

Figure S7. QC analytical HPLC of peptide **2**

(conditions, gradient, and column can be found in the General Experimental section). Retention time 12.67 min.

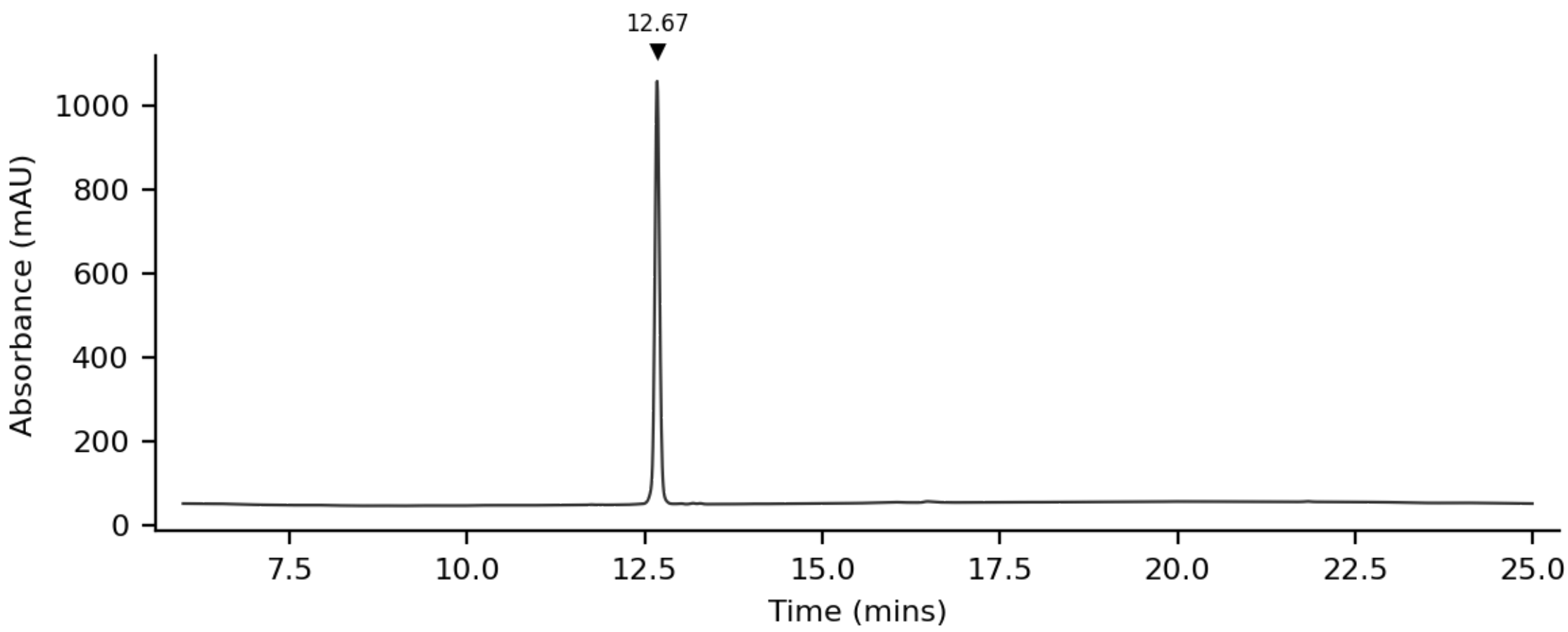

Synthesis of oxidised peptide 3

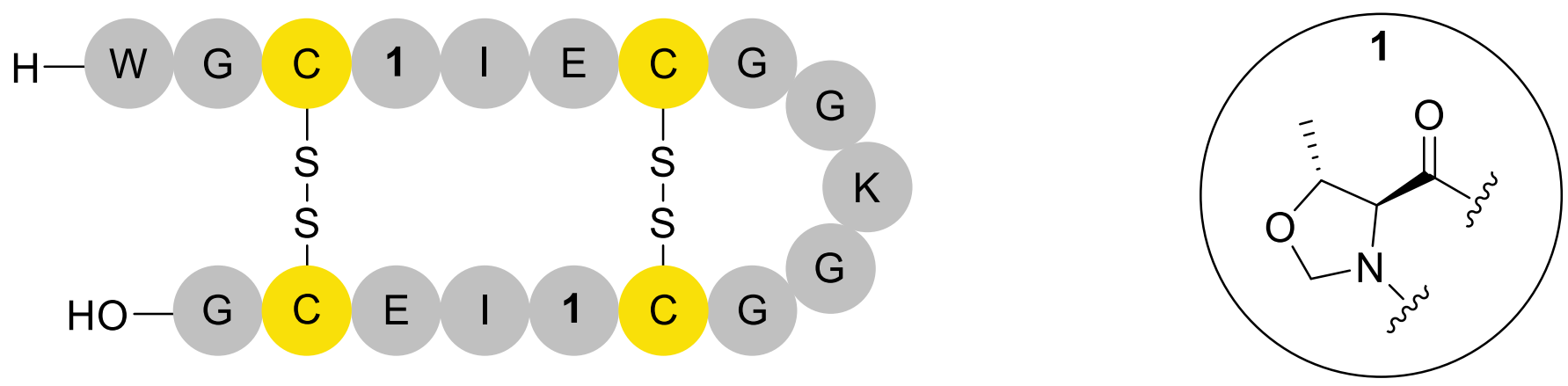

3

100 mM potassium phosphate monobasic buffer (pH=7.4) containing 0.5 mM oxidised glutathione (GSSG) was added into a low adsorption centrifuge tube, followed by the addition of peptide **2** to a final concentration of 100 μM. After 4 hours of reaction in a shaker at 37ºC, the reaction was quenched by 10% HPO_3. Finally, the reaction was monitored by HPLC and the oxidation products were analysed by mass spectrometry.

Mass Analysis: product has an elemental composition of $C_{73}H_{108}N_{20}O_{25}S_4$. Calculated exact mass of product is 1792.6677 Da.

HRMS: *m/z* calculated for $C_{73}H_{109}N_{20}O_{25}S_4$ ($[M+H]^+$) 1793.6756, LC-MS analysis observed 1793.6750. Isotopic spacing of the $C_{75}H_{112}N_{20}O_{25}S_4$ ($[M+2H]^{2+}$) ion shows definitively that this species is doubly charged.

Analytical HPLC trace: R_t for major product 12.674 mins (86.5%), R_t for minor product 12.674 mins (13.5%), (5-95% B over 30 mins, λ = 214 nm).

Figure S8. QC MS of isolated peptide **3**.

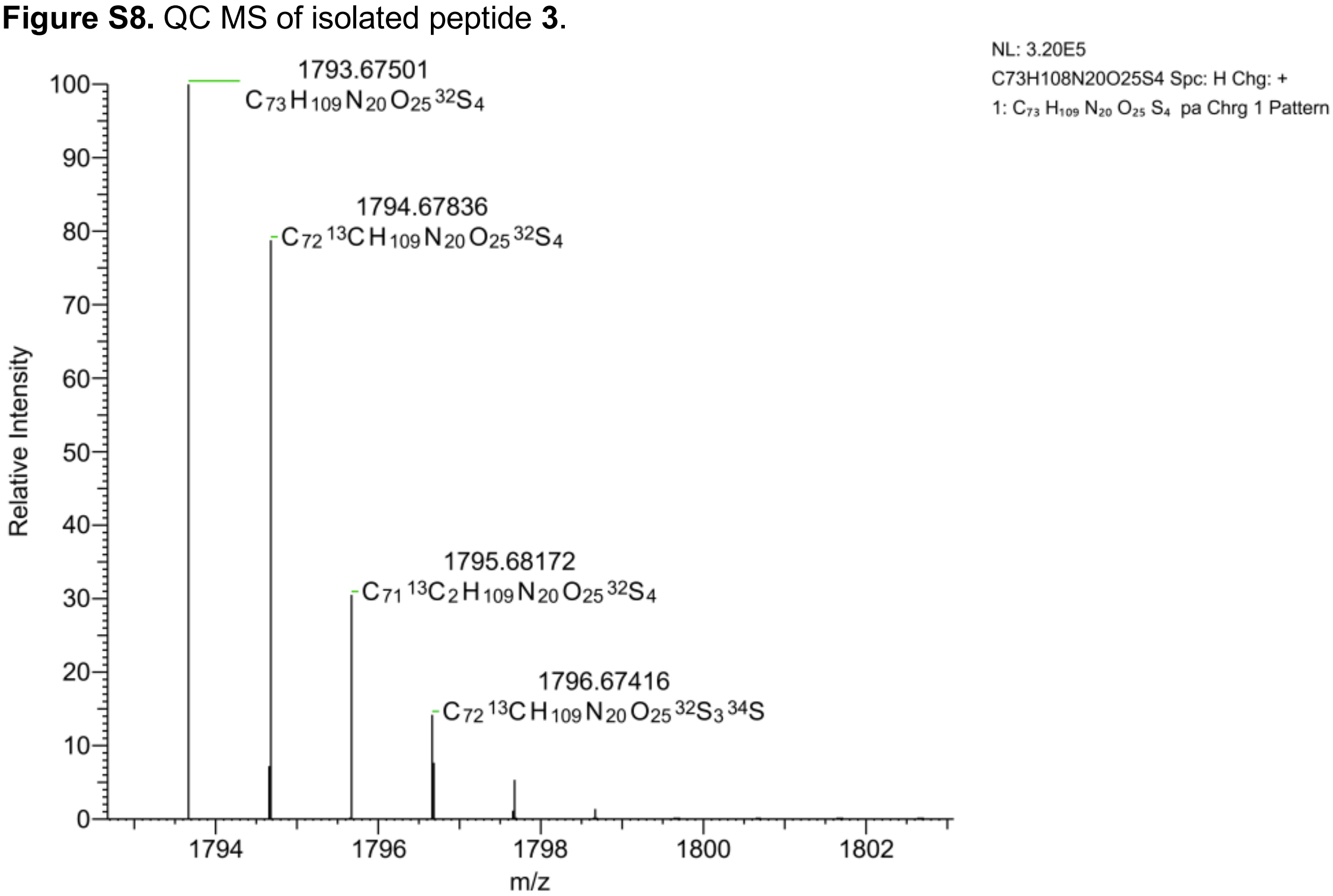

Figure S9. Analytical HPLC traces to show the oxidation profile of peptide **2** and isolation of its major oxidative product, peptide **3**.

Peptide **3** is produced with an 86% yield from peptide **2**.

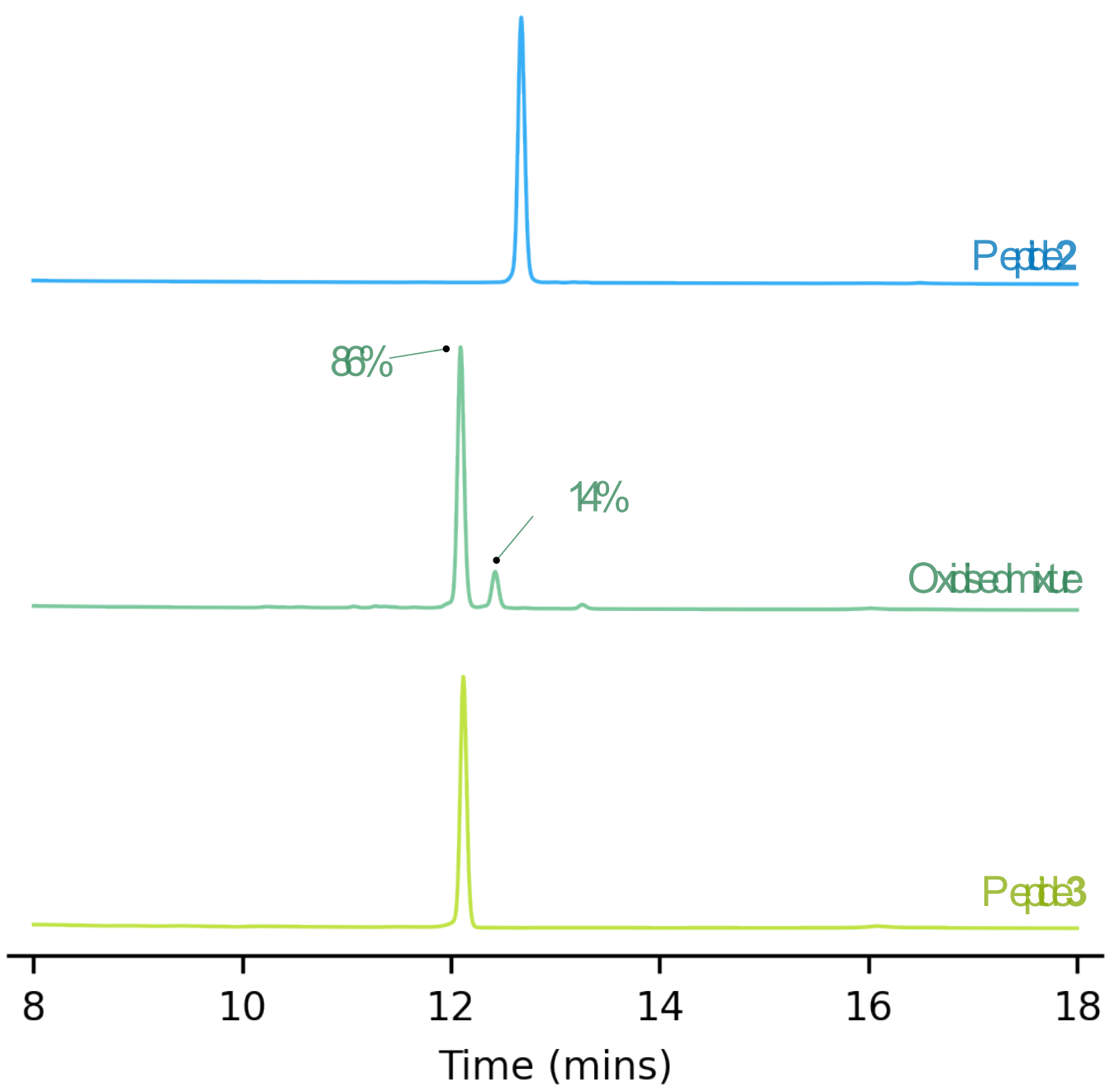

Further information for IM-CA-IM-MS experiments

Table S3. Detailed cyclic sequence for slice-CA experiments conducted on peptide **3**.

Step	Function	Time (ms)		Pre-array gradient (V)	Pre-array bias (V)	Offset (V)	Array T-wave height (V)
		Conformer A	Conformer C				
**1**	Inject	10.00	10.00	85	70	45	2 (forwards)
**2**	Separate	33.00	33.00	85	70	70	35 (sideways)
**3**	Eject	1.50	8.00	85	70	45	25 (forwards)
**4**	Eject to Pre Store	0.75	0.75	45	40	55	25 (backwards)
**5**	Hold and Eject	12.75	6.25	45	40	45	25 (forwards)
**6**	Reinject from Pre Store	10.00	10.00	85 (+x V)	70	45	2 (forwards)
**7**	Separate	33.00	33.00	85	70	45	35 (sideways)
**8**	Detect and Acquire	-	-	-	-	-	-

Gaussian peak fitting residuals

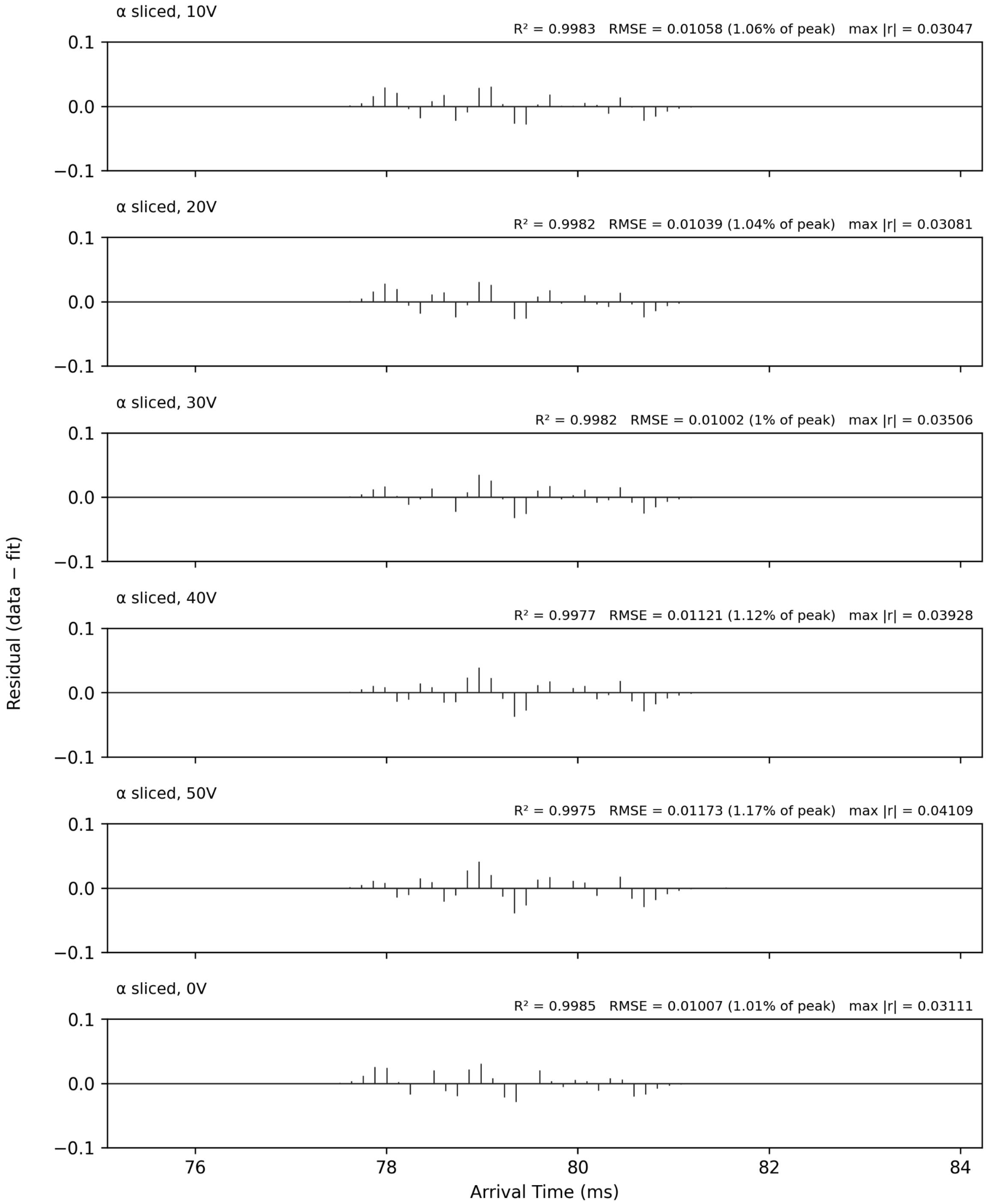

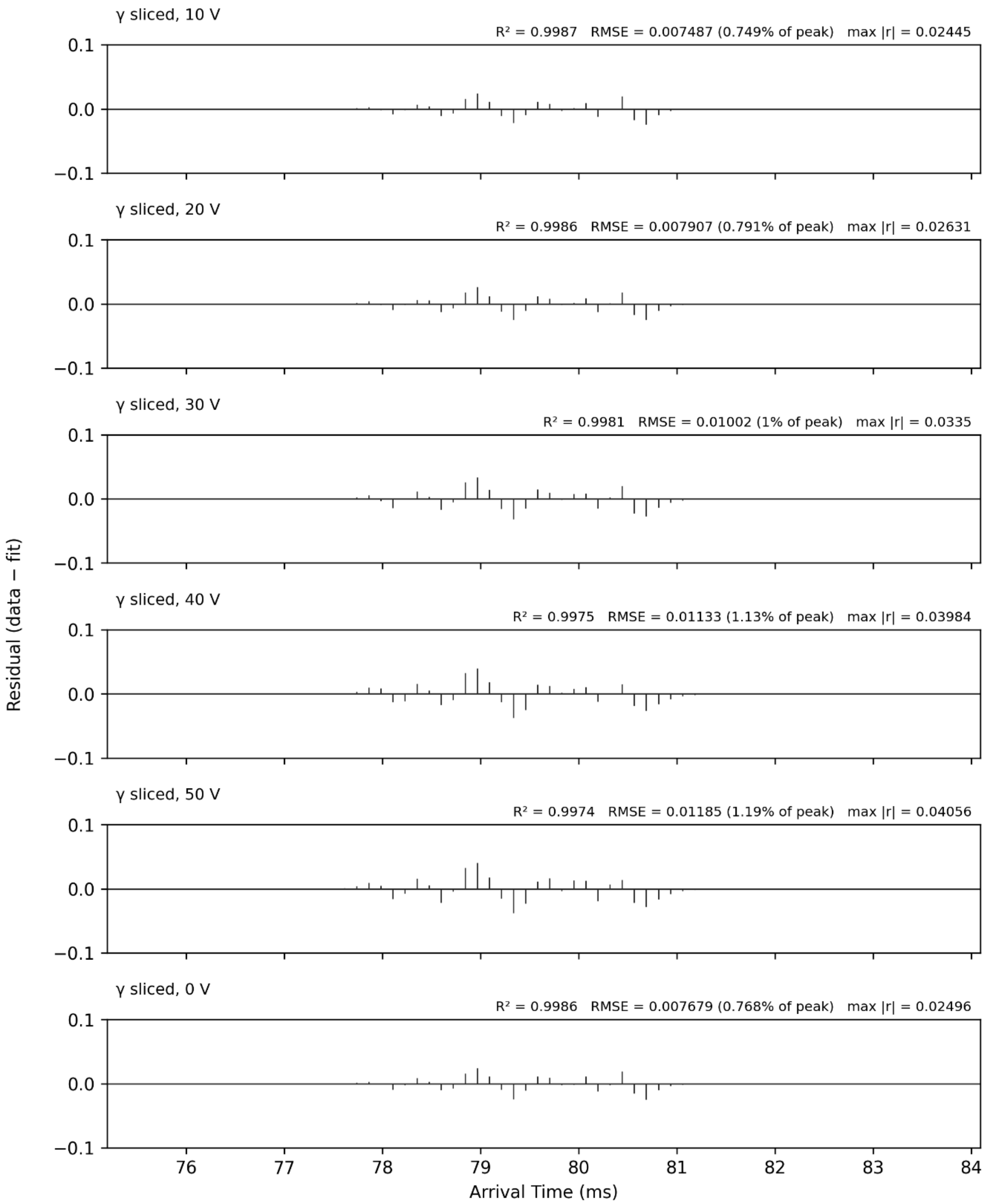

Figure S10. Residual analysis of Gaussian deconvolution across collision-voltage conditions.

Residuals are shown for composite Gaussian fits to the ion-mobility arrival-time distributions acquired at 0-50 V for both sliced α and γ conformers (Top and bottom, respectively). All resolved conformational components were included in each fit, and residuals were calculated pointwise as the observed normalized intensity minus the fitted intensity. Positive and negative values therefore indicate experimental signal above and below the composite fit, respectively. The coefficient of determination (R^2), root-mean-square residual (RMSE), RMSE expressed as a percentage of the normalized peak maximum, and maximum absolute residual $\max|r|$ are reported for each condition. Identical arrival-time and residual-intensity scales are used throughout to permit direct comparison of fit quality between voltages.
